# Factors that impede women’s contraceptive care needs during and immediately after incarceration in New South Wales, Australian. Qualitative perspectives of professional staff working with incarcerated women

**DOI:** 10.1371/journal.pone.0355277

**Published:** 2026-08-31

**Authors:** Helene Smith, Mandy Wilson, Basil Donovan, Jocelyn Jones, Tony Butler, Paul Simpson

**Affiliations:** 1 School of Population Health, University of New South Wales, Sydney, New South Wales, Australia; 2 Kirby Institute, University of New South Wales, Sydney, New South Wales, Australia; 3 Kurongkurl Katitjin, Centre for Indigenous Australian Education & Research, Edith Cowan University, Perth, Western Australia, Australia; 4 National Drug Research Institute, Curtin University, Perth, Western Australia, Australia; University of North Carolina at Chapel Hill, UNITED STATES OF AMERICA

## Abstract

**Introduction:**

Women with a history of incarceration report higher rates of pregnancy and abortion, lower contraception use and poorer birth outcomes compared to other women in the community. This suggests family-planning and reproductive-health needs that are not well served by current models of care. Despite this, little is known about how contraception is provided within Australian carceral settings, or the structural barriers experienced by professional staff working in these environments. We report findings from a qualitative study conducted in New South Wales (NSW), Australia, describing professional staff’s views of factors that support or impede women’s contraceptive care needs both during and immediately after incarceration.

**Methods:**

We conducted semi-structured interviews with n = 12 professionals working in NSW, who either provide healthcare services or individual case management to women during incarceration or in the immediate post-release period. Data was analysed thematically, initially guided by the Vulnerable Populations Behaviour Model and adjusted iteratively as analysis developed.

**Results:**

Participants described contraceptive needs and preferences as shaped by 1) a state of interrupted fertility caused by incarceration, and 2) post-release instability and intersecting demands, which impeded access to community family planning. Areas of high need were characterised by 1) accessible, reversible and non-invasive contraception options, and 2) thoughtful counselling. Structural factors impeding contraception service access during incarceration included: 1) service priorities 2) poor overall health service integration, and 3) inequitable funding models.

**Conclusion:**

Structural constraints during incarceration, staff attitudes and women’s complex post-release circumstances, limit equitable contraception care, underscoring the need to address barriers at system, clinician and patient levels.

## Introduction

Australian women with a history of incarceration have higher rates of pregnancies, abortions [1–3], and report lower rates of contraceptive use [4] compared to women in the community. In addition, babies born to women with a history of incarceration are more likely to be preterm, have lower birth weights and have a 50% greater risk of spending five or more days in a neonatal/ intensive care unit compared to community controls [5]. Children of women with a history of imprisonment also experience more than double the rates of infant mortality compared with children of mothers with no incarceration record [6]. All of which indicates that existing models of care do not adequately meet the family planning and reproductive health needs of women with a history of incarceration.

The provision of comprehensive contraception care, which includes not just the ability to access and insert appropriate and reversible modern contraception, but also be able to easily remove, has long been regarded as a crucial service in ensuring economic empowerment for women along with reducing maternal and child morbidity and mortality [7,8]. However, it is also increasingly recognised that for many women, pregnancy planning and intention can be fluid concepts [9–15] and that critical human rights and Reproductive Justice principles need to be prioritised when these services are provided to socially or economically disadvantaged women. This is particularly critical for long-acting reversible contraception (LARC), because initiation and discontinuation depend on clinician-controlled insertion and removal, increasing women’s reliance on providers and heightening the risk that access constraints or power imbalances can undermine truly voluntary, non-coercive contraceptive decision-making.

In the context of Australia, population, qualitative and primary-care research over the past decade indicates that access to reversible contraception (including oral contraceptive pills, condoms, emergency contraception, and clinician-dependent methods such as Intrauterine Devices (IUDs) and implants) is shaped by interacting barriers at individual, service and system levels. Population data indicates that the pill and condoms remain the most commonly used methods, with implants and IUDs accounting for a much smaller share of contraceptive use [16–18]. Across studies, commonly reported access barriers include out-of-pocket costs and gap fees, geographic availability and limited service capacity (particularly in rural and regional areas), consultation time constraints and variability in counselling quality, workforce and training limitations affecting timely provision of insertion and removal, confidentiality concerns and stigma (especially for younger women); the cultural safety and acceptability of care for Aboriginal and Torres Strait Islander women, and pharmacy- and service-level constraints affecting timely access to emergency contraception and privacy at the point of care [19–21].

Contraceptive care constraints can also interact with life stage, relationships and preferences, shaping which methods feel feasible, acceptable and sustainable over time. For example, qualitative work with young women suggests that method choice reflects knowledge of available options, cultural norms, past experiences and concerns about side-effects, and preferences around control and autonomy, which may contribute to continued use of the pill or condoms and less frequent reassessment of preferences over time [22]. Furthermore, studies of contraceptive consultations in general practice describe time constraints and the influence of clinicians’ own preferences, which can limit shared decision-making across the full range of methods [23].

These constraints can shape not only access, but also the balance of power and information within contraceptive encounters, affecting whether choices are fully informed and freely made. This makes human rights and Reproductive Justice principles, especially non-judgmental care, shared decision-making, and support to discontinue or change methods, essential to high-quality contraception care [24–26].

Reproductive Justice advocates recognise that women’s ability to enact their true reproductive choices are often hampered by intersecting and systematic oppressions. These can be driven by legal and institutional structures, as well as demographic factors such as race or socio-economic status [27–30]. These issues are compounded within a carceral setting, which is inherently coercive, constrains autonomy, and heightens risks of reproductive coercion and compromised voluntariness [31]. The prison environment also interrupts and systematically separates families through remand, short sentences, incarceration far from home, restrictive visiting regimes, and increased child-protection involvement [6,32], directly conflicting with a core Reproductive Justice tenet: the right to parent in safe and sustainable communities free from violence [29,33,34]

In Australia, the carceral setting is over-represented by women from economically and socially disadvantaged communities, including a large proportion of Aboriginal and Torres Strait Islander women who are imprisoned at more than 21 times the rate of non-Indigenous women and constitute a disproportionate share of women in custody [35]. These patterns reflect the colonial origins, as well as ongoing forms of colonial surveillance within the Australian criminal-legal system, with specific, intergenerational harms for Aboriginal and Torres Strait mothers and caregivers that are central to any Reproductive Justice considerations [36,37].

Women in Australia with a history of incarceration are also more likely to have experienced significant trauma [1] and substance use disorder histories [38], compared to women in the community. In custody, the patient-, – and system-level constraints described above may be intensified by service-access barriers such as long-wait times and limited continuity of care [39–41]. These barriers may also be compounded by routine carceral disruptions and administrative processes [42], as well as relational and cultural safety constraints, including judgmental and racist treatment by custodial staff and distrust of authority figures [43–45]. Low awareness of available health services can also further limit access [39].

Comparable evidence from the United States and Canada indicates substantial unmet need and uneven availability of family-planning services in custody with most facilities lacking robust onsite contraception programs, policies described as inconsistent, and access to clinician-dependent methods is frequently constrained [46,47]. There is also evidence that non-coercive provision of IUDs, implants and broader family planning services is both feasible and acceptable when implemented [46,48,49].

No studies have considered whether these issues are mirrored in the Australian carceral system, and the transferability of these models to Australia is uncertain because the health-system interfaces that underpin contraception access differ across jurisdictions. In Australia, correctional health services are funded and delivered by state/territory governments, and people in prison are generally ineligible to use Commonwealth-funded schemes such as Medicare or the Pharmaceutical Benefits Scheme such as Medicare and the Pharmaceutical Benefits Scheme [50, 51]. By contrast, in the United States there is no universal health insurance scheme, and Medicaid generally cannot be billed for most routine care provided in prisons and jails (with limited exceptions, such as inpatient hospital care), leaving correctional authorities and contracted providers to fund and deliver care during custody [52]. In Canada, while medically necessary care is publicly funded in the community through provincial/territorial plans, health services for people in federal custody are delivered through federal corrections rather than provincial health insurance [53]. In the Australian community, Medicare and the PBS scheme provide free or highly subsidised health services and medicines that underpin affordable contraception access, including General (medical) Practitioner (GP) consultations for counselling and prescribing, as well as subsidised insertions and removals of IUDs and implants [54].

In this article, we report findings from a qualitative study conducted with a sample of professionals working in New South Wales (NSW), who either provide reproductive healthcare services to women during incarceration or in the immediate post-release period. Specifically, we examined how staff understand and respond to the contraceptive needs of women who experience imprisonment and release. Our objective was to describe these professionals’ views on the factors that may support or impede women’s contraceptive care needs during and immediately after incarceration, including how staff perceived women’s fertility intentions and experiences of pregnancy in shaping contraceptive decision-making.

## Methods

### Study design

Between August 2022 and July 2023, a qualitative study was conducted, where 12 participants were recruited to complete a one-hour semi-structured interview with one of the authors (HS).

Both interview guides and the coding framework were informed by the Behavioural Model for Vulnerable Populations [55], which offers an empirically supported framework to understand the combination of multi-level factors, termed “predisposing characteristics” (that is, demographic and social structure characteristics of individuals accessing the service), “need factors” (perceptions and evaluated needs of special relevance to vulnerable populations) and “enabling factors” (either individual or organisational characteristics that may influence access to family planning services). The framework was selected due to its past use in prison settings [56] and in identifying key factors that may influence the use of contraception in socially and economically disadvantaged communities [57]. We report this qualitative study in accordance with the Consolidated Criteria for Reporting Qualitative Research (COREQ) 32-item checklist [58] (S1 Checklist)

### Study setting & description of current contraception policies

In 2023, 768 adult women were held in custody in Australia. Nearly half had received and were serving a sentence (49.9%), with the remaining unsentenced and being held on remand. Forty-one percent were Aboriginal or Torres Strait Islander [59]. Women held in custody in NSW fall under the care of Corrective Services NSW, with the Justice Health and Forensic Mental Health Service (hereon Justice Health NSW) responsible for the provision of health services. This service fully funds all healthcare services in lieu of the availability of Medicare (Australia’s universal healthcare insurance scheme), as well as the provision of medications and commodities (such as the contraceptive pill and IUDs), which would normally be available through the Pharmaceutical Benefits Scheme (PBS), neither of which are available to incarcerated persons [60].

Justice Health NSW policies state that all incarcerated persons should have access to equivalent services that they would be able to receive in the community. This includes the provision or replacement of contraceptive devices, including the oral contraceptive pill, emergency contraception, injectables, implants and IUDs[62]. Aboriginal and Torres Strait Islander women should be offered these services by a registered Aboriginal health practitioner, who provides clinical services and patient care with a focus on culturally safe practice and who facilitates relationships between Aboriginal and Torres Strait Islander patients and other health practitioners and services [61,62]. Contraception services can be provided by either the women’s health nurse, midwives as part of post-partum care or a general practitioner (GP). To access any health service, women must submit a written request, which can be handed either directly to nursing staff, correctional staff or placed in a box which is then directly accessed and triaged by nursing staff, who will then assign to the appropriate provider pending availability and perceived urgency. Upon release, women can access the same community-based reproductive health services available to the broader population, and may also be supported by specialised post-release organisations that provide tailored, case-by-case assistance to link women to appropriate care where needed.

### Participant recruitment

Through a combination of web searches, professional networks, and a snowball sampling approach, we identified staff employed by either Justice Health NSW who provide reproductive health services to women during incarceration, or community-based organisations involved in providing health services or individual case-management for women in the immediate post-release period (n = 12). Potential participants were contacted by email with an information sheet about the study, and upon indicating interest in the study, and having questions answered, were asked to provide verbal informed consent prior to participation in the study. Following consent, participants were requested to forward the study information onto other potentially interested colleagues.

### Data collection

Each participant was interviewed for approximately 1 hour on Zoom/Teams and guided by a semi-structured interview schedule which focused on organisational and individual priorities, services provided, demand for services, barriers to service delivery, individual patient characteristics that impact service delivery, and personal views around incarcerated women’s access to reproductive health services. This analysis specifically examined only the data collected in regards to contraception.

All interviews were conducted by HS, a PhD student with prior experience in qualitative research on incarceration and reproductive health who had no clinical or managerial relationship with participants. Before and after interviews, the researcher discussed their assumptions about prison health care and contraception with the wider study team to support reflexive practice during data collection and analysis.

### Data analysis

After the data were collected, recordings were transcribed and uploaded to NVIVO12 (QSR, Australia) for analysis. Data was indexed by themes as well as by demographic information including the job description of each participant. Using both inductive and deductive approaches, a thematic framework was developed to identify key choices, challenges and relational conditions required for women to have their reproductive healthcare needs met. As part of the analysis, a 6-phase protocol for thematic analysis (informed by Braun & Clarke) [63] was applied.

During analysis the Behavioural Model for Vulnerable Populations was used as a sensitising framework, initially organising codes into “predisposing characteristics”, “need factors” and “enabling factors” as described by the original model [55]. As coding progressed, it became clear that the data mapped unevenly onto the “enabling” domain as participants overwhelmingly described organisational and system-level conditions that constrained rather than facilitated women’s access to contraception (for example, service priorities, fragmented models of care and funding arrangements), and very few clearly “enabling” or facilitative features were identified. To avoid implying that these factors operated positively, while still remaining faithful to the underlying model in which enabling resources can function as barriers or facilitators, we re-labelled this third analytic domain as “structural factors that influence access”. In the Results, themes within this domain therefore correspond to the model’s enabling conditions at individual and organisational levels (for example, service configuration, workforce, referral pathways and financing), but are described using terminology that more accurately reflects participants’ emphasis on structural barriers in this setting rather than on the presence of supportive enabling resources.

Following analysis, the themes identified and data supporting the themes were reviewed by two additional members of the study team (MW and PS) to consider the suitability of the findings as well as individual biases in the interpretation of the results. Furthermore, a draft version of the findings was shared with participants who had indicated they would like to review sensitive information provided. To ensure confidentiality due to the small sample size, participants were labelled into broad categories of GP, nurse and non-government organisation (NGO) worker.

In line with the terminology used by participants, we use the term “women” to describe the population of interest. We acknowledge that not all people who can become pregnant identify as women, and that our findings may also be relevant to pregnancy-capable people of other genders.

### Ethics approvals

This study was approved by University of New South Wales (#HC210826), Justice Health & Forensic Mental Health Network (#G1044/21) and Aboriginal Health & Medical Research Council (#1947/22) Human Research Ethics Committees.(S2 Ethics)

## Results

### Participant characteristics

Twelve individuals were identified and consented to participate in the study. Seven participants worked for Justice Health NSW as clinical service providers within the correctional health system, and five worked with NGOs. The NGOs represented a mix of clinical providers of family planning services for the general community including recently incarcerated women, and people who were directly involved in the case-management of women either during or immediately after their incarceration. These participants worked for a mix of organisations including an Aboriginal community-controlled health organisation, an advocacy group and an organisation that specifically provided case-worker support services for previously incarcerated women.

Half of the participants (n = 6) were GPs, two (16%) were nurses employed in either a midwife or women’s health role, with the remainder having non-clinical roles including advocacy, casework and clinic management. All participants had been involved in the care of incarcerated or recently incarcerated women for at least five years, two participants identified as Aboriginal and/or Torres Strait Islander, and one participant had previously been incarcerated. None of the participants were attached to a specific prison but rather worked across several facilities or supported women who had come from a variety of locations across NSW.

### Findings

Seven themes were identified and are presented here according to the three main fields of enquiry. They include: 1) predisposing characteristics of incarcerated women, 2) need factors for incarcerated women, and 3) structural factors that influence access to contraception.

**Fig 1:**
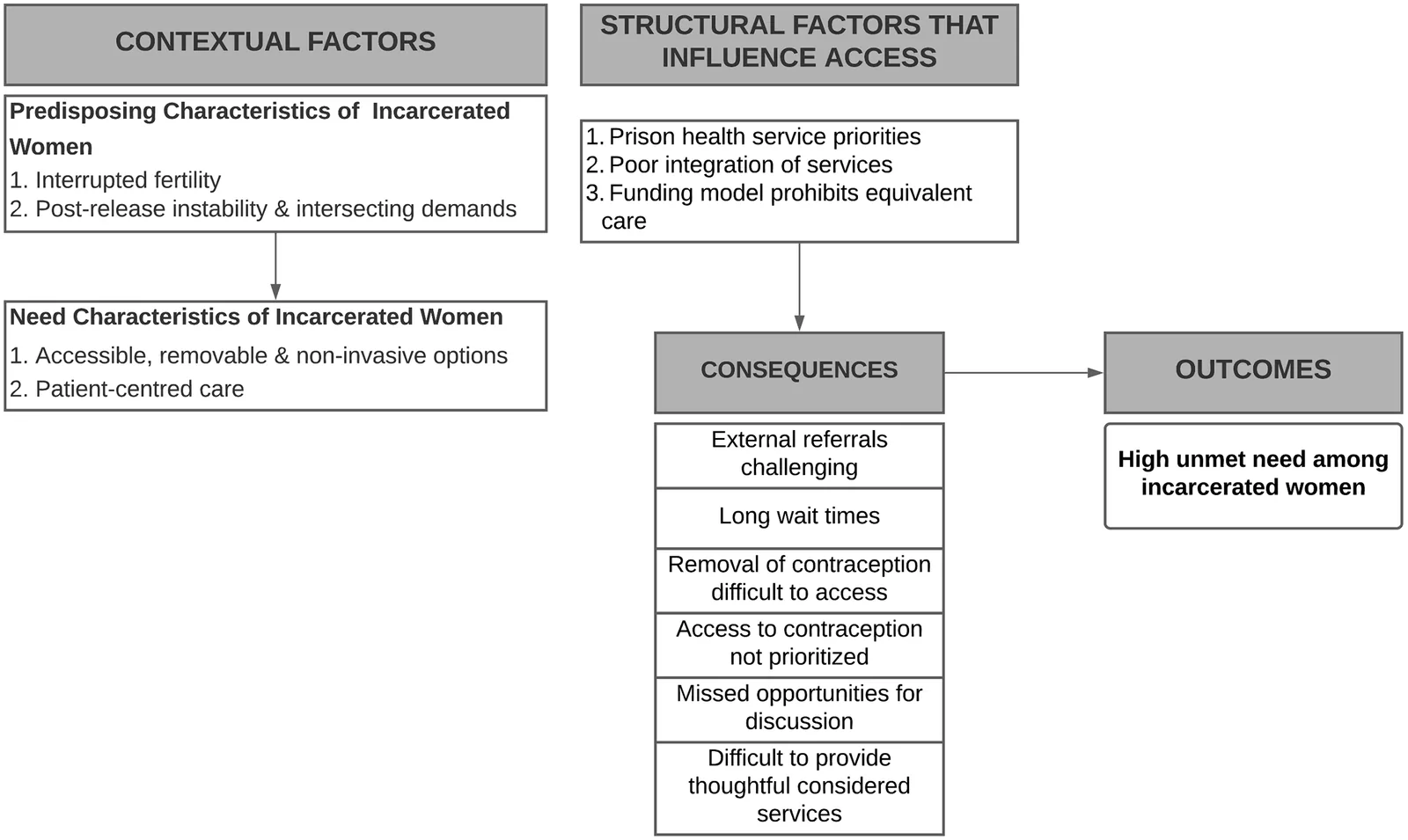
Factors professionals working with incarcerated women in NSW believe influence incarcerated women’s contraceptive needs and preferences during incarceration.

We present these themes and their consequences on the outcome of interest, access to contraception, in Fig 1.

### Predisposing characteristics of incarcerated women

#### Interrupted fertility.

Many participants observed that incarceration could heighten women’s concerns about future fertility, particularly in the context of separation from children, menstrual disruption during custody, and a broader loss of control over reproductive decision-making.

*“What struck me a lot is how frustrated women who’ve been in prison can feel in terms of their fertility… a lot of them have already had children, but often they’re kind of, you know, maybe estranged from their children, or just really enjoy having children and don’t want to feel like they’re missing out […]I wonder whether that feeling of not necessarily being in control in here really heightens those thoughts”*

GP

Furthermore, several clinical staff believed that the nature of the prison environment created a heightened anxiety amongst women regarding their fertility opportunities post-release. One GP described these feelings below in the context of women’s periods stopping during their incarceration.

*“I see a lot of women whose periods have changed when they’ve come into prison, and often they’ll stop. And so, for some people, you know, that can sometimes feel like a convenient thing, but for most of the women, they find it quite a fearsome thing, that they no longer have their periods. And they can see that as a sign of potentially being damaged by the prison environment and their fertility for the future being damaged.”*

GP

As a result of these sentiments, many clinical staff had observed that conversations about pregnancy prevention and associated contraception methods were often quite nuanced, with a need to balance the clinician’s belief that a woman might be better suited to longer term contraception such as an IUD, and the patient’s desire to not completely halt the potential for future children in the near future.

*“I think the long-acting sort of contraceptions are often a very good choice because it’s just such a difficult time for people when they’re released from prison… So, you know, having just sort of long-acting contraception to help you not have to worry about that is important, I think. But that said, it’s also not what the women themselves want to do… They don’t know what’s coming and they want to leave space for children”*

GP

#### Post-release instability and intersecting demands.

Participants who worked with women after release noted that this period was often characterised by instability and uncertainty, fuelled by unstable housing, substance use issues and/or returning to violent relationships.

*“The chaos of, especially that kind of initial post release period, in terms of relationships, in terms of drug use, in terms of homelessness, and a lot of women kind of, you know, sort of having kind of housing in a sex for favour type sort of set up, like so much”*

NGO STAFF

They observed that this often caused women to struggle to engage with community health services at release, making it difficult to access contraception.

*“So, there’s other challenges, the mental health, the communication, you know, unable to communicate their needs… And then, you know, because we’ve got a lot of women that are barred from services as well. So, but that’s, you know, as a result of not getting their needs met, not being able to communicate their needs, and then just sort of giving up because they’re like, nah, I can’t cope with this”.*

NGO STAFF

NGO staff remarked that they often saw the consequences of poor access to contraception post-release with frequently needing to support recently released women with accessing abortion services. They further remarked that these women often needed support accessing abortions later in pregnancy, which were far more costly and invasive procedures. Staff believed that this was due to women not having access to contraception in the first place, financial issues with terminating a pregnancy early but also that women reported being turned away from clinics earlier on in their pregnancy and felt so judged or stigmatised around their choices to terminate that they delayed seeking appropriate services.

*“So sometimes they will go to* [THE CLINIC]*, to, to explore their options and say this is what they want to do. And they don’t feel that people are actually hearing them when they say they want a termination, and they don’t want to have this child. So, they’re like, ‘No, it’s a big decision. You go back and think about it and come back’... and they don’t come back”.*

NGO STAFF

Some NGO staff interpreted this high demand for abortion services in the post-release period as reflecting unmet contraceptive need among some newly released women.

*“If someone is wanting contraception, then the ideal place to have that done, if it’s their choice, is in custody before they are released. So then, when it’s done and dusted, it’s happening, it’s in place. Because once they get out […], it’s really hard”*

NGO STAFF

#### Need characteristics of incarcerated women.

*Accessible, reversible and non-invasive options:* Participants observed high demand for reversible methods, particularly those perceived as more flexible or feasible in the context of women’s changing circumstances after release, including the contraceptive pill, short-acting injectables such as Depo-Provera®, and clinician-dependent methods such as implants and IUDs.

While most participants, both NGO workers and clinical staff, believed that hormonal or non-hormonal IUDs may be more suitable given many women of the competing priorities that women faced when released, they acknowledged that this was not as popular among the women they worked with, who often wanted more flexible and less invasive methods.

*“We encourage them to go for long-term contraception rather than, you know, adhering to a pill, anything that requires a regime or rather you being adherent to it.”*

GP

*“They* [WOMEN] *like the idea of taking a pill that they can stop and start themselves and don’t want that lack of autonomy and, you know, having something you have to have taken out by a doctor, and just access sometimes, it’s just the issue, you know, you’d like an IUD, but there’s no one to take it out.”*

GP

Depo shots were in particularly high demand, despite both staff and NGO workers feeling that these might not be the most logistically appropriate for women leading unstable lives as it required regular engagement with a health service. Despite this reservation, they attributed its popularity to being less invasive and short-acting.

*“There’s a lot of depo, which is what I’ve seen too with women who are coming through drug health services, they would put them on the depo. But like it’s, it’s chaotic for those women as well, because they’ve got to come back for an injection every 12 weeks.”*

NURSE

#### Patient-centred care.

All participants recognised that a key component of providing appropriate contraception to women during their incarceration included patient-centred care, centred on thoughtful counselling, where women’s individual fertility aspirations post-release and their past experiences needed to be considered. Women with sexual assault histories often needed additional counselling when considering any sort of vaginal insertion, both regarding whether they felt comfortable with this type of method and preparing them for the insertion procedure.

*“We’re talking about a cohort of women who have generally had very high experiences of sexualized violence, domestic violence, childhood sexual assault […] They’ve been stripped, searched on a regular basis […]. People are in kind of that trauma response [..] And so going through a process of having something like that [VAGINALLY INSERTED] can be actually quite terrifying.”*

NGO STAFF

*“If you’ve been sexually assaulted, maybe kind of like, you’re not actually that comfortable with people putting things inside your vagina…”*

GP

Clinical staff also highlighted the importance of being able to refer incarcerated women to specialist services in the community for complex insertions and removals (needed due to anatomical issues, high levels of distress or predisposing medical issues such as epilepsy).

*“I am not going to put a Mirena*^*®*^ *in someone who’s going to jump off the bed on me. Sometimes the women are a bit like that […} I just don’t want to do harm. So, on, you know, if I don’t think that they’re suitable to have it done with me in the jail, then I will send them out.”*

NURSE

Furthermore, considerations around who should provide the counselling or services, for example, an Aboriginal health worker for Aboriginal and/or Torres Strait Islander women, also needed to be considered.

*“I’ve heard [INCARCERATED] women talk about […] if they have a First Nations person there and an older First Nations woman there, who’s speaking to them, who’s from outside and just from the community, there is less fear of judgement, there is less fear of discrimination. And there’s less, there is more sense of this person will be okay, if I share this, they’re not going to have a viewpoint of me, this isn’t coming from a practice of trying to annihilate me, disappear me. Judge me, view me in a different way. Because of who I am, aware of my cultural connection.”*

NGO STAFF

Nearly all staff recognised that their ability to implement such thoughtful and considered counselling was challenging to deliver in the context of where they worked. The factors that contribute to this are discussed under *Structural Factors that Influence Access* below.

### Structural Factors that Influence Access

#### Prison health service priorities.

Most participants believed that the correctional health system was designed and funded to focus on acute and complex needs. This included a focus on severe mental health problems, substance use issues, and infectious diseases treatment. One of the consequences of this was that health issues such as contraception support often fell through the cracks.

*“People with complex needs end up getting looked after, but maybe the less complex ones might, you know, have less priority and less involvement,”*

GP

Whilst several Justice Health NSW staff felt that there had been vast improvements to preventative screening services such as mammograms and Pap-smears, several staff reflected that there did appear to be a blind spot amongst some staff when providing comprehensive contraceptive services to women. One nurse described other nurses being genuinely bemused as to why contraception should be prioritised at all.

*“I think that lack of support from other staff is very frustrating […] We’re trying to do what’s best for them, so that they can get on with their life in the community. I mean, sometimes they say, ‘Oh, why? Why are you doing contraception? […] they don’t need it in jail’. And then we have to explain…. Well, we’re doing this for when they’re released.”*

NURSE

Several NGO staff suggested that this attitude, in part, related to prioritising immediate health needs rather than considering needs within a continuum of care, both during and after a woman’s incarceration. They also suggested it was due to a de-gendering of women during their incarceration.

*“Corrective Services has a viewpoint that their responsibility is to keep people safe and contained for the duration of their time […] ‘This is our responsibility. Our responsibility isn’t after. So, this is all I’m going to look out for’. So that is one reason I think that it’s totally missed, because they don’t see it as their responsibility.”*

NGO STAFF

*“People aren’t thought about so much in terms of whether or not they want to have babies or whether or not they want to have contraception, they’re thought about as a prisoner […] a kind of minimising of those other aspects of women’s lives when somebody goes to prison […] it’s [REPRODUCTIVE HEALTH] so far down the list in terms of how people are thinking about women in prison, and how people are thinking about access more broadly, and how people are kind of humanising or dehumanising the women that end up in in prison.”*

NGO STAFF

As a result, most participants agreed that women often experienced challenges accessing appropriate contraception services. This included difficulty arranging external services for complex inserts and removals, long-wait times to meet with the women’s health nurse and most notably, challenges in having contraception removed. According to several participants, factors impacting contraception removal included – the lack of prioritisation of the service, and the perceived “appropriateness” of a woman to be able to have children by staff.

*“It’s always worried me a bit, because, you know, women will sort of come in and, you know, you can see they’ve got long acting, contraception already in, they’ll tell you, they want it out. Because they want to fall pregnant when they leave prison. But, you know, you can see that, in fact that they’re in a chaotic state stage, and probably not making a good choice to have the contraception out […] Nevertheless, of course, it does become their choice, but I wonder whether providers might be declining to take them out?[…] [WOMEN ARE] feeling unsupported in wanting to have it taken out”*

GP

#### Poor integration of services.

Staff involved in the provision of contraceptive services recognised that that their ability to provide thoughtful and considered counselling around contraception was hampered by structural factors such as a siloed health delivery model, poor integration with community services, particularly in relation to Aboriginal health services, and weak pre-release planning systems.

Services were often provided as targeted interventions rather than an integrated system, which staff noted resulted in lost opportunities for discussions around reproductive health. Several GPs noted that this siloed structure was also creating backlogs for the women’s health nurse, of which there was only one. They described any “female-focused service” being referred to her, creating unnecessarily long waiting lists and pressure on the women’s health nurse to quickly turn around patients, encumbering her ability to have detailed conversations with patients.

*“It’s all* [REQUEST FOR CONTRACEPTION] *devolved to the women’s health nurse. We’ve only got, I think, one women’s health nurse to the whole of New South Wales. So, you know, she’s absolutely marvellous, but obviously only human, so she’s not going to be able to capture everyone.”*

GP

Poor integration of family planning services at release meant that opportunities for thoughtful and considered discussion were often missed. This was driven by myriad challenges that impacted all pre-release planning including difficulties to predict departures, chaotic systems and poorly completed paperwork. One GP noted that the extent of these issues varied from centre to centre and referrals related to contraception did take place in smaller facilities, with smaller staff to incarcerated women ratios, combined with a more stable population allowed for more thoughtful planning.

*“It depends so much on the context. So, I used to work at a smaller prison, which is now at least temporarily closed, which was minimum security and had a strong focus on preparing people for the community. And it really was quite a routine thing for the primary care nursing staff to find out whether, you know, at release, contraception was required, but now, for example, I work in a Remand Centre, which is obviously much more chaotic, with some people released from there and some people in there for a long time.[…] I can well imagine that many women are released without having had that conversation”*

GP

NGO staff noted that their observations of some women leaving prison without contraception, falling pregnant and then requiring support to access abortion services, suggested poor access to contraception as part of pre-release planning, noting that women on remand were at a heightened risk of not having any pre-release support.

*“I don’t think it is something that is prepared for on release […] women do not come out with contraception. So, it’s definitely an area that’s missed [..] many women are on remand… for that population, there isn’t really anything much in the way of pre-release planning”*

NGO STAFF

Several participants described a chronic lack of Aboriginal health workers, who were perceived as critical players in providing culturally appropriate care.

*“They don’t have enough of an Aboriginal workforce that you can just see an Aboriginal person when you want. Yeah, it’s a big, big issue”*

GP

Aboriginal workforce shortages were generally attributed to ongoing challenges in making Justice Health NSW roles attractive to Aboriginal healthcare providers, combined with poor integration with community services.

*“There are always open positions for Aboriginal health practitioners, they [ARE] constantly attempting to recruit staff to those positions, and there’s a big turnover… There are many reasons for that. […] I think that a lot of [Aboriginal] people with that sort of qualification are probably quite keen to work in their communities”*

GP

#### Funding model prohibits equivalent care.

Most participants recognised that the suite of contraception types available through the correctional health system was not comparable to what was available in the community. For example, both copper IUDs and certain brands of contraceptive pill (that offered less side-effects) were generally not available due to their higher costs.

*“We are missing out on the copper IUDs, because I think they’re too expensive for the jail”*

GP

*“You’re actually constrained by what I think might be an appropriate pill […]. I do a lot of, like a very thoughtful you know, considered approach, based on the woman and her circumstances. So, some of the pills that I like aren’t available. They would be if we could access the PBS”*

NURSE

Furthermore, participants noted that the lack of access to services and medicines funded by both Medicare and the PBS, impacted their ability provide equivalent services to what was available in the community. This was particularly noted when providing specific Aboriginal health services.

For example, the funding arrangement limited opportunities for local Aboriginal Medical Services to provide services that were available in the community. One Aboriginal participant noted that if the systems were truly equivalent, community-based Aboriginal health services would be better empowered to provide a comprehensive continuum of care to incarcerated women, allowing for more personalised considerations around reproductive and contraception choices.

*“We [AMSs] should be resourced to be able to keep in contact with our women. If they get transferred from say regional or remote area into the incarcerated centres within an urban area, then we could communicate with a local AMS that this this lady has been incarcerated, and we need the AMS in that location to be able to go and provide ongoing care… but there’s no funding mechanism to do that”*

NGO STAFF

Several NGO staff believed that the solution lay in the provision of more innovative and flexible ways of providers being able to claim Medicare rebates whilst providing services to inmates.

NGO participants also suggested that inadequate funding for AMSs to provide justice-based services was in part due to a lack of formal mandate, but also a general lack of interest from AMSs in supporting incarcerated people in the community.

*“Ya know, for our people, especially in the AMS sector lane, we talk about working with our people from life to death, right? But when they go into jail, we don’t go anywhere near them... I don’t think there’s a major focus on it. And that’s scary.”*

NGO STAFF

## Discussion

To our knowledge, this is the first Australian qualitative study to examine the views of health care and social services professionals on factors that support or impede women’s contraceptive care needs during and immediately after incarceration. Some of the issues identified, such as provider reluctance to perform IUD removals mirror issues identified in the community [23,64,65], however they appear to be magnified in a secure setting where individual autonomy and personal self-efficacy are severely limited due to the structural constraints of the prison health-system. Given the complex and sensitive circumstances many women experience during their incarceration, including trauma histories, housing instability, violent relationships, substance use, and nuanced fertility intentions, our findings suggests that contraceptive care requires greater tailoring and care.

We identified several factors that require further consideration. Perhaps most importantly, the *“predisposing characteristics”* of incarcerated women highlight a conundrum regarding reproductive health and autonomy, and the complexities that many service providers must navigate. Service providers want to encourage women to initiate long-term methods such as IUDs, so as to protect them from unintended pregnancies in the context of substance use, unstable housing and competing priorities on release, yet our findings show that incarceration can simultaneously intensify some women’s desire for future children and amplify fears about infertility. Similar concerns have been documented amongst incarcerated women in North America [43,66,67] but to our knowledge this is the first time they have been described in an Australian sample of healthcare and social welfare professionals who work directly with these women.

In this light, the accounts by NGO workers of women attempting to access abortion services but receiving little engagement or practical assistance from their providers, is particularly revealing. When contrasted with providers’ reluctance to remove implants or IUDs at women’s request, this points to a troubling pattern in which health services appear more responsive to institutional priorities to prevent pregnancy than to women’s expressed reproductive goals. While unwillingness to remove LARC constrains women’s medium- to long-term reproductive choices, failure to act on a request for abortion has immediate and profound consequences for women who are forced to continue an unwanted pregnancy. These dynamics resonate with work on reproductive coercion and abuse, both in Australia and elsewhere, in which provider inaction or discouragement can function as a form of control over women’s fertility [68,69].

Our findings also sit within a broader structural context in which, women in the community who are actively trying to avoid or terminate a pregnancy often face poor access to services and, in some cases, explicit pressure to continue a pregnancy. This aligns with Australian research documenting economic barriers relating to high out-of-pocket costs for abortion, particularly in regional and remote areas [70], as well as reports of reproductive coercion during the counselling phases prior to abortion care [69]. For women who have recently been incarcerated, these financial, geographic and organisational barriers intersect with stigma and system-level control over their bodies, amplifying inequities in reproductive choice [47,68]. Because this study did not examine abortion access directly, we cannot draw detailed conclusions beyond the implications of constrained contraception access, however, these findings underscore the need for dedicated research on abortion pathways and barriers for women before and after release.

Our findings underscore the need for holistic and intensive case-management support for women both during and after incarceration, including proactive assistance to navigate contraception, fertility concerns and abortion care, reflecting the distinct and uniquely layered reproductive health needs of this population compared with women in the general community. Unfortunately, as many participants pointed out, adequate resources do not currently exist to support this level of care, with this gap evident across several domains and themes.

Firstly, ‘*Structural Factors that Influence Access’* highlighted a focus on acute care needs, including substance use disorders, infectious disease and mental health, which whilst important and needed, can result in a lack of resources available to support routine, holistic and patient-focused care during incarceration. The implications of this, such as the emergence of medical emergencies that could have been prevented if given attention earlier, has been extensively reported [40,45,71], its consequences on services such as contraception however, have received less attention. Participants also emphasised that these constraints are intensified by the high proportion of women on remand, short and unpredictable sentences, as well as unanticipated transfers, which create constant churn and limit both women’s ability to request care and providers’ ability to complete planned contraception, abortion or other preventive care. These structural barriers have been documented in previous Australian prison-health research [41,42,72]. Whilst prison-health surveys demonstrate the need for focusing on such acute needs [38,72], our findings demonstrate the importance of ensuring that the provision of these services do not come at the expense of more future-focused planning of services that may prove to be highly valuable to women, particularly in the post-release period.

Secondly, whilst certain female specific services, such as mammograms, Pap-smear services and antenatal care were reported to be well supported, our theme related to *‘prison health* *service priorities’* highlighted that several participants expressed concerns around the ‘de-gendering’ of women’s needs during incarceration, which was reflected with limited staffing numbers available to deal with women’s health issues. Whilst a sizable body of literature has raised concerns around this problem, this has more often been discussed in relation to prison systems and service models historically developed around much larger male prison populations, due to their much higher numbers within carceral settings [73–75]. Our findings suggest a deeper de-humanising issue, where any sense of women being considered as fully formed human beings, with intentions and hopes that go beyond their current incarceration, is removed. Whilst this is certainly also the case for incarcerated men, for women, such hopes can include future pregnancy intentions, or the desire to avoid pregnancy. Ignoring this facet of women’s lives makes it impossible to abide by Reproductive Justice principles in this setting, and, more fundamentally, it underscores that fully realising Reproductive Justice within carceral settings is not achievable without addressing incarceration itself as the central constraint. However, this does not negate the imperative to pursue meaningful, rights-affirming improvements in contraceptive support for women who are incarcerated now.

Thirdly, our theme *‘funding model prohibits equivalent care’* suggest that the equivalence of care principle [76] is not being upheld regarding the provision of contraception for incarcerated women. Incarcerated women are unable to access the same range of contraception options available through the PBS, nor are they able to access integrated models of care that have been developed in the community in Medicare funded services. This is particularly important for the care of Aboriginal and Torres Strait Islander women who are unable to access health services from the community that have been modelled on culturally safe practices and are delivered and informed by Aboriginal community-controlled healthcare principles and delivery models.

In addition, participants from Aboriginal community-controlled organisations (ACCHOs) described a striking gap between the sector’s commitment to providing “whole-of-life” care and the practical exclusion of people during periods of incarceration, which one NGO worker characterised as “scary”. This disconnect echoes previous work showing that ACCHOs and AMSs are only patchily engaged in providing primary health care to people in prison or during the immediate post-release period, largely due to limited funding, unclear mandate and restricted access to custodial settings [77,78]. Recent national guidance similarly emphasises that Aboriginal and Torres Strait Islander people’s health needs are best served when prison health services build formal partnerships with ACCHOs to support continuity of culturally safe care on release yet notes that such arrangements remain inconsistently implemented [79]. Our data suggest that these system-level gaps also shape access to contraceptive and reproductive health care, reinforcing calls for dedicated resourcing and clearer mandate for justice-focused work within the AMS/ACCHO sector [80].

Fourthly, our theme *‘service priorities’,* highlighted healthcare provider perceptions around women’s reproductive intentions may be impeding women’s ability to have devices removed. Whilst nearly all participants in this study expressed desires to ensure that women in their care were able to express individual choice and agency in their reproductive desires, they acknowledged that these views could be complicated. Participants acknowledged that concerns around a woman’s ability to support a child, given complex histories involving drug use, homelessness, mental health and/or previous child removals, could lead a provider to create structural barriers to the removal of devices. In an environment where individual agency is minimised, such actions essentially result in completely blocking access to a particular service. Women who are perceived by society as ‘unfit reproducers’, due to economic poverty, social status, (mental) health status, race, and/or life history have historically been at risk of reproductive coercion and nonconsensual sterilisations [27,81]. The nature of many women’s profiles during incarceration makes them particularly vulnerable to approaches that encourage these oppressions either consciously or subconsciously. Whilst forced sterilisation, particularly in the US prison systems have received high levels of attention and criticism [82–84], more subtle forms of reproductive coercion, resulting in women being unable to enact their contraceptive choices, including contraception removal, clearly remain. These require thoughtful consideration with the carceral setting.

These patterns can also be understood through stratified reproduction, where social, economic and racial hierarchies determine whose fertility is supported and whose is constrained [85]. In our data, women with intersecting disadvantages, including criminalisation, substance use, child protection involvement and, for many, Aboriginal and Torres Strait Islander identity, were frequently constructed as “risky” mothers, making pregnancy prevention an institutional priority over women’s own reproductive goals. Staff reluctance to remove IUDs and rods, limited abortion access and the neglect of contraception in release planning all illustrate how such hierarchies shape practice and constrain reproductive autonomy [34,85].

Findings from this study have several implications. Firstly, the anecdotal implication that women are leaving prison with a high un-met need for contraception, as suggested by several stakeholder participants, needs to be quantified. Secondly, the findings as outlined here, which represents the views of service providers, need to be triangulated with the views of incarcerated women. Both are currently being explored as part of the 2^nd^ Sexual Health and Attitudes of Australian Prisoners (SHAAP-2) study which is currently being implemented in NSW. Thirdly, these findings provide additional justification as to the importance of incarcerated persons being able to access Medicare services. Finally, as the rate of female incarceration continues to increase both globally [86] and in Australia [87], our study highlights the importance of ensuring that gender-specific services, such as contraception, are provided in a thoughtful and empowering way. More broadly, if the carceral system is genuinely committed to “rehabilitation”, contraception and broader reproductive health care should be understood as part of the resources people need to live healthy, self-directed lives on return to the community, rather than being confined to meeting only immediate needs while incarcerated [88]. Whilst there is increasing recognition that key concepts of reproductive justice, such as individual agency in managing reproductive choices, is fundamentally irreconcilable with existing in a state of incarceration [89,90], attempts must be made to improve the mechanisms by which these services are delivered within current incarceration settings.

Our findings should be considered in light of several study limitations. Our sample comprised of a small sample size of 12, however, the limited attention that contraceptive services were reported as receiving in the carceral setting, reflects that only a small number of health-care workers are involved in the provision of these services. Our extensive snow-balling strategy indicates that the primary players involved in the provision of contraception were included and, furthermore, the inclusion of NGO staff, who support women outside of Justice-Health gave strength to the perspectives provided by the small sample. However, we should note that we observed two key groups, whose perspectives are not included. Firstly, despite numerous outreach efforts, we received high rates of refusal to participate from nursing staff unless they had a specific reproductive health role. This speaks to the challenges discussed by individuals who did particulate in having contraception recognised as a standard health-care services rather than a specialised service. Secondly, during the study, it became clear that the views and practices of corrective services staff would be an important area to explore. However, due to the constraints on the research placed by institutional approvals and PhD candidature timelines, this examination was not possible. We therefore recommend that future research investigate the perspectives and experiences of these stakeholders. Finally, whilst many facets of this study will have replicability in other settings, it is important to note that all participants worked in NSW and for publicly funded prisons. Privately managed prisons may have unique features which merit further exploration.

## Conclusion

The availability of comprehensive and patient-centred family planning services is a human right. Not providing such services represent a missed opportunity in supporting women in the justice system who have distinct and unique needs that may become exacerbated immediately after release. Our findings highlight the complexities of providing these services within a carceral setting, including the effects of structural constraints, service priorities, and staff biases. Key structural factors that need to be considered, to provide appropriate contraceptive services, include considerations around service priorities, addressing staff biases, better integration of contraception services into other services and better funding models to support equivalence of care principles.

## Supporting information

S1 Checklist COREQ 32-Item checklist. (PDF)
