# Supplementary material for: Factors that impede women’s contraceptive care needs during and immediately after incarceration in New South Wales, Australian. Qualitative perspectives of professional staff working with incarcerated women
Source: PLoS One. 2026 Aug 31;21(8):e0355277. doi: 10.1371/journal.pone.0355277 (PMC13528980; doi:10.1371/journal.pone.0355277)
Supplement: Supplementary file 1 [file pone.0355277.s001.pdf]

COREQ (COnsolidated criteria for REporting Qualitative research) Checklist

A checklist of items that should be included in reports of qualitative research. You must report the page number in your manuscript where you consider each of the items listed in this checklist. If you have not included this information, either revise your manuscript accordingly before submitting or note N/A.

Topic	Item No.	Guide Questions/Description	Reported on Page No.
**Domain 1: Research team and reflexivity**			
Personal characteristics			
Interviewer/facilitator	1	Which author/s conducted the interview or focus group?	HS
Credentials	2	What were the researcher's credentials? E.g. PhD, MD	PHD candidate – MPH
Occupation	3	What was their occupation at the time of the study?	Student
Gender	4	Was the researcher male or female?	Female
Experience and training	5	What experience or training did the researcher have?	Extensive qualitative interview experience including with justice-involved populations and correctional staff in a number of settings
Relationship with Participants			
Relationship established	6	Was a relationship established prior to study commencement?	Participants had emailed with the interviewer prior
Participant knowledge of the interviewer	7	What did the participants know about the researcher? e.g. personal goals, reasons for doing the research	They knew the interviewer was from UNSW and part of the SHAAP-2 study.
Interviewer characteristics	8	What characteristics were reported about the inter viewer/facilitator? e.g. Bias, assumptions, reasons and interests in the research topic	PHD background and history of working in reproductive health
**Domain 2: Study design**			
Theoretical framework			
Methodological orientation and Theory	9	What methodological orientation was stated to underpin the study? e.g. grounded theory, discourse analysis, ethnography, phenomenology, content analysis	Thematic Analysis informed by Braun & Clarke
Participant selection			

Sampling	10	How were participants selected? e.g. purposive, convenience,<br>consecutive, snowball	Purposive and snowball
Method of approach	11	How were participants approached? e.g. face-to-face, telephone, mail,<br>Email	Email
Sample size	12	How many participants were in the study?	12
Non-participation	13	How many people refused to participate or dropped out? Reasons?	2 stated they didn't work in reproductive health, 1 declined to participate as she needed approval from her employer (corrective services) which was outside the scope of the study
Setting			
Setting of data collection	14	Where was the data collected? e.g. home, clinic, workplace	Online
Presence of non-participants	15	Was anyone else present besides the participants and researchers?	No
Description of sample	16	What are the important characteristics of the sample? e.g. demographic<br>data, date	Professionals working wither as case workers or directly providing reproductive health services to women with a history of incarceration
Data collection			
Interview guide	17	Were questions, prompts, guides provided by the authors? Was it pilot<br>tested?	Yes<br>Adjustments were made after the first interview
Repeat interviews	18	Were repeat inter views carried out? If yes, how many?	NO
Audio/visual recording	19	Did the research use audio or visual recording to collect the data?	Yes
Field notes	20	Were field notes made during and/or after the inter view or focus group?	Yes
Duration	21	What was the duration of the inter views or focus group?	Up to 1 hour
Data saturation	22	Was data saturation discussed?	Yes
Transcripts returned	23	Were transcripts returned to participants for comment and/or	No

Topic	Item No.	Guide Questions/Description	Reported on Page No.
		correction?	
**Domain 3: analysis and findings**			
Data analysis			

			8
Number of data coders	24	How many data coders coded the data?	1 (checked by 2 others)
Description of the coding tree	25	Did authors provide a description of the coding tree?	No
Derivation of themes	26	Were themes identified in advance or derived from the data?	Derived from the data
Software	27	What software, if applicable, was used to manage the data?	Nvivo
Participant checking	28	Did participants provide feedback on the findings?	No
Reporting			
Quotations presented	29	Were participant quotations presented to illustrate the themes/findings?<br>Was each quotation identified? e.g. participant number	Yes<br>Identified by type of participant
Data and findings consistent	30	Was there consistency between the data presented and the findings?	Yes
Clarity of major themes	31	Were major themes clearly presented in the findings?	Yes
Clarity of minor themes	32	Is there a description of diverse cases or discussion of minor themes?	Yes – within the major themes

Developed from: Tong A, Sainsbury P, Craig J. Consolidated criteria for reporting qualitative research (COREQ): a 32-item checklist for interviews and focus groups. *International Journal for Quality in Health Care*. 2007. Volume 19, Number 6: pp. 349 – 357

Once you have completed this checklist, please save a copy and upload it as part of your submission. DO NOT include this checklist as part of the main manuscript document. It must be uploaded as a separate file.
