# Supplementary material for: Factors that impede women’s contraceptive care needs during and immediately after incarceration in New South Wales, Australian. Qualitative perspectives of professional staff working with incarcerated women
Source: PLoS One. 2026 Aug 31;21(8):e0355277. doi: 10.1371/journal.pone.0355277 (PMC13528980; doi:10.1371/journal.pone.0355277)
Supplement: Supplementary file 2 [file pone.0355277.s002.pdf]

Thank you for the opportunity to review this paper on an under-researched and important topic. In general, I think the reproductive justice framework needs more consideration and thought to be fully realized. I have listed additional comments and suggestions below.

General note: Paper needs thorough copy editing for grammar, particularly use of commas when not appropriate.

Abstract
Line 32-33 - I suggest re-wording your description of the sample since actually only 8 participants were clinicians. I certainly see the value of interviewing other people who play a role in the provision of healthcare, but would not consider them to be “providing healthcare services”. Perhaps professionals working in family planning? Or similar wording.

Introduction
Line 65 - Can you state some of the birth outcomes?
Line 68 - And including access to abortion?
Line 80-81 - I would suggest stronger language here. Beyond questions of coercion becoming precarious, I would argue the prison environment is inherently coercive. If you are using an RJ framing here, would also suggest a few lines on how the prison environment interrupts and systematically separates families, in direct conflict with RJ tenet to be able to parent in safe and sustainable communities free from violence. I also think this introduction needs some reference to the over-incarceration of Aboriginal and Torres Strait Islander women in Australia, and the colonial context of the prison system, which has huge implications for reproductive justice.
Line 102-105 - In light of the lack of studies in the Australian context, can you look to analogous contexts internationally where there has been work done on family planning services in prison? E.g. Martha Paynter (Canada), Carolyn Sufrin (USA). You could say studies conducted in other carceral settings have found xxx… however an Australian study is needed because of (unique context of Medicare, PBS etc.).

Methods
Line 147 - Suggest briefly defining an Aboriginal health worker for non-Australian audience who may not have an equivalent role in their context
Line 149-150 - Do they submit the request directly to nursing staff? Or is it first submitted to a guard, who then submits to nursing staff? This has implications for patient confidentiality and ethics (if first submitted to officer/guard, which has been noted in Canadian & American studies)
Line 171 - Since you state using a theoretical framework of reproductive justice, how was this considered or included in your analysis process? The RJ framing gets a little lost past the introduction.

Results
General note: I suggest referring to the terminology guide published by the Australian National Network of Incarcerated Women and Girls which does not recommend the term ‘justice-involved’: [https://thenationalnetwork.com.au/abolition-resources/](https://thenationalnetwork.com.au/abolition-resources/)
Line 195-197 - Not sure this is needed.

Note that line numbering stops after the figure. Please add line numbering in future submission to facilitate review.
Page 14 - Please refer to clinical language guidelines re:abortion and avoid 'later term abortion': https://www.acog.org/contact/media-center/abortion-language-guide or https://www.childrenbychoice.org.au/wp-content/uploads/2023/01/Language-Guide-2022.pdf for example
Page 14- Wow, this quote is very powerful and implies forced pregnancy. This seems to me to go beyond not being able to access timely services and is actually referring to outright denial of care. Perhaps stronger language needed to frame this.
Page 15- I don't love the framing of women's "chaotic lifestyles". It feels a little stigmatizing. While technically true, might be more appropriate to explain how women are experiencing intersecting demands and determinants of health and access upon release from prison. This removes the focus from individual behaviour choices/lifestyle…
Page 18 - Re-word to "one of the consequences on this….etc" sentence sounds incomplete as currently written.

Discussion
Line 2 - What is contraceptive support? This term hasn't been used before. Simpler to say contraception?
See note above re: justice-involved
Also, not sure this statement is true as currently written. Sutherland et al. (2015) did technically consider factors that may have led to low uptake in their sample. Similarly, Heggie Noonan et al. (https://www.sciencedirect.com/science/article/pii/S0277539524001572) explored how geographic factors might impede access to abortion in prison in Australia. Your study is certainly the first (as far as I can find!) to qualitatively examine in-depth multiple factors from the perspective of family planning professionals. Suggest re-wording.
Page 25- Again suggest moving away from the chaotic lifestyle framing and instead detailing some of the intense challenges faced by women upon release from prison.
Page 25- I would argue reproductive justice cannot be achieved in a prison setting. I do find the RJ framing quite weak throughout (mentioned in intro but not integrated into methodology as a theoretical framework). I would remove this statement, or expand on it. I think your study has identified numerous factors that make RJ not possible in a prison setting, such as a lack of autonomy, delay or outright denial of care, lack of culturally informed services, etc….
Page 25 - "Several factors were identified requiring further consideration" Incomplete sentence
Page 25 second paragraph - and perhaps speaking to incarcerated women about what outcomes and services matter to them, in this future-focused planning?
Page 26 first paragraph- Yes, this does make RJ impossible in this setting. Suggest using this stronger language above where noted.
Page 26 first paragraph - can you cite a study with incarcerated men to support this statement?
Page 28 - Re: inability to interview corrective services NSW staff, are there plans to do this in the future?
