# Supplementary material for: Factors that impede women’s contraceptive care needs during and immediately after incarceration in New South Wales, Australian. Qualitative perspectives of professional staff working with incarcerated women
Source: PLoS One. 2026 Aug 31;21(8):e0355277. doi: 10.1371/journal.pone.0355277 (PMC13528980; doi:10.1371/journal.pone.0355277)
Supplement: Supplementary file 3 [file pone.0355277.s006.pdf]

4 May 2026

RE: Revisions for PONE-D-25-46905

To the Editors of *PLOS ONE,*

We thank-you very much for your recent feedback for the journal article entitled: "*Why are you doing contraception? ... They don't need it in jail". Service provider views on women's access to contraception in Australian prisons*", which you provided on the 7th November 2025.

We have reviewed all feedback and made the recommended changes as requested. These are listed below, alongside a detailed outline of how each point has been addressed.

Please note that the line references below refer to the line reference in the TRACKED CHANGES document.

Academic editor / journal requirements:

1. *"Please ensure that your manuscript meets PLOS ONE's formatting requirements. PLOS ONE has a manuscript format similar to the sample papers listed below"*
Thank-you for providing these examples. We have checked formatting against the PLOS ONE title/affiliations and main-body samples and updated the title page and manuscript layout accordingly

2. *"Your ethics statement should only appear in the Methods section and not elsewhere. Please ensure your ethics statement only appears in the methods section as is required."*
We have reviewed the full manuscript and ensured that ethics information is stated in the Methods section only under Ethics Approvals [LINE 280]

3. *"We note that you have indicated that there are restrictions to data access. Please include the following information in the Data Availability statement in the submission form accordingly"*
We have updated the Data Availability Statement in the PLOS submission form to address restrictions, what data are restricted, and where requests may be sent

4. *"Please include captions for your Supporting Information files. This information should be included in the submission form on the Attach Files page. In addition, please ensure that your file names use the following format and are cited properly in the manuscript: S1 File, S1 Table, S1 Fig, etc."*
We have ensured Supporting Information files use the required naming conventions for the figure provided. and that it has an appropriate caption.

5. *"We note that there is identifying data in the Supporting Information file provided. Please remove identifying information and resubmit. Note that all columns in Supporting Information will appear in the published file. Therefore, please ensure that no columns are hidden as all hidden columns will appear in the published file."*
We reviewed the Supporting Information files for identifying content and hidden columns. The files visible to us do not appear to contain identifying participant information beyond investigator names on ethics documents. If the editorial comment refers to another supporting

file, we would be grateful for clarification so we can address it directly. Should this comment be referring to another file, please do let us know so we can address this.

6. *"If the reviewer comments include a recommendation to cite additional works, please review and consider such recommendations, and incorporate them into the manuscript if appropriate."*
   We have reviewed all citation recommendations and updated references where relevant and appropriate throughout the manuscript

Reviewer #1

ABSTRACT:

1. I suggest re-wording your description of the sample to note not all participants were *clinicians. It currently suggests all participants are "provide healthcare services to women". You can broaden this a little. Only 8 participants were clinicians, I suggest similar wording."*
   Thank-you for pointing this out. We have revised both the Abstract and Methods to accurately reflect the composition of the sample and avoid implying that all participants were clinicians. Specifically, we revised the wording to make clear that the sample included both clinical and non-clinical participants working during incarceration and/or the immediate post-release period, including healthcare providers and participants in case-management roles. This change is reflected in the Abstract Methods [LINES 36-38], and the distribution of roles (including that 8 participants were clinicians) is clarified in the Participant characteristics section [LINES 286-297].

2. *Can you state some of the birth outcomes?*
   We have specified the adverse birth outcomes rather than referring to "poorer birth outcomes" generally; the Introduction now explicitly notes increased likelihood of preterm birth, low birth weight, and neonatal/intensive care stay, alongside infant mortality differences. [LINES 56-62]

3. *"And including access to abortion?"*
   We have clarified in the introduction that women with a history of incarceration report accessing abortion services more than women in the general community. [LINES 56-57] As there is scant evidence on how easy it is for these same women to access abortion in the community, we have kept the point in the introduction to where the existing research lies: reporting higher number. We then address the lack of evidence and comparable evidence around women from lower socio-economic backgrounds in the discussion, in light of the findings where some participants believed that women struggled to access timely abortion care in the community [LINES 662-678].

4. *"I would suggest stronger language here. Beyond questions of coercion becoming precarious, I would argue the prison environment is inherently coercive. If you are using an RJ framing here, would also suggest a few lines on how the prison environment interrupts and systematically separates families, in direct conflict with RJ tenet to be able to parent in safe and sustainable communities free from violence. I also think this introduction needs some reference to the over-incarceration of Aboriginal and Torres Strait Islander women in*

Australia, and the colonial context of the prison system, which has huge implications for reproductive justice"
Thank-you for highlighting this. We have revised the introduction text to state explicitly that carceral settings are inherently coercive and constrain autonomy, and we have added text on systematic family separation/conflict with the RJ tenet of parenting in safe/sustainable communities. We have also added a paragraph on over-incarceration of Aboriginal and Torres Strait Islander women and the colonial context and implications for RJ. [LINES 112-126]

5. *"In light of the lack of studies in the Australian context, can you look to analogous contexts internationally where there has been work done on family planning services in prison? E.g. Martha Paynter (Canada), Carolyn Sufrin (USA). You could say studies conducted in other carceral settings have found xxx… however an Australian study is needed because of (unique context of Medicare, PBS etc.)"*
   We have rewritten this section so the logic is explicit rather than implied. Specifically, we have:
   - clarified the evidence gap (limited Australian custody-focused contraception research) [LINES 128-129]
   - explained why international models (primarily North American) may not transfer directly, and stated the key structural reason for this difference in Australia, primarily due to different funding mechanisms that fundamentally impact how services are delivered [LINES 130-146]

METHODS:

6. *"Suggest briefly defining an Aboriginal health worker for non-Australian audience who may not have an equivalent role in their context"*
   We have added a brief definition of an Aboriginal Health Worker/Practitioner in the Methods where this role is first mentioned, describing it as an Indigenous-specific health workforce role that provides culturally safe care and supports engagement, navigation and continuity between clients and health services (often including health promotion, advocacy and liaison). [LINES 203-207].

7. *"Do they submit the request directly to nursing staff? Or is it first submitted to a guard, who then submits to nursing staff? This has implications for patient confidentiality and ethics (if first submitted to officer/guard, which has been noted in Canadian & American studies)"*
   We have clarified the actual pathway for requesting health services in custody so readers can see when confidentiality may be protected versus when it may be compromised. In the Study Setting/Access description, we now specify that women must submit a written request and that this request may be (a) handed directly to nursing staff, (b) handed to correctional staff, or (c) placed in a request box accessed and triaged directly by nursing staff. We have included this detail because the involvement of correctional staff in request submission has clear implications for privacy and aligns with confidentiality concerns raised in international prison-health literature. [LINES 208-212].

8. *"Since you state using a theoretical framework of reproductive justice, how was this considered or included in your analysis process? The RJ framing gets a little lost past the introduction"*

Thank-you for this observation. In the Introduction, we have clarified that Reproductive Justice (RJ) is not used as our primary theoretical analysis framework, instead we draw on RJ principles to frame the broader context of discussion of reproductive health service provision in carceral settings, and to highlight the inherent contradictions this creates for women's reproductive autonomy [LINES 109-126]. For the analytical framework, we now state more explicitly in the Methods that our analysis was initially guided by the Vulnerable Populations Behaviour Model and then iteratively adapted as themes developed, including a specific adaptation to better capture organisational/system constraints (reframing "enabling factors" as "structural factors influencing access"). [LINES 179-180 and 266-276]. In the Discussion, we have strengthened integration of RJ by explicitly linking our findings on constrained autonomy, gatekeeping, and discontinuity across custody-community transitions to RJ principles, and we have adopted stronger language about the impossibility of fully realising RJ under the constraints described. [LINES 718-720 and 732-733].

RESULTS:

9. *"General note: I suggest referring to the terminology guide published by the Australian National Network of Incarcerated Women and Girls which does not recommend the term 'justice-involved': https://thenationalnetwork.com.au/abolition-resources/"*
Thank-you for providing this resource. We have adapted our language throughout to describe either women with a history of incarceration, or who are incarcerated. This has been done throughout the manuscript.

10. *"Note that line numbering stops after the figure. Please add line numbering in future submission to facilitate review."*
We apologise for this, this has now been rectified throughout the manuscript

11. *"Please refer to clinical language guidelines re:abortion and avoid 'later term abortion': https://www.acog.org/contact/media-center/abortion-language-guide or https://www.childrenbychoice.org.au/wp-content/uploads/2023/01/Language-Guide-2022.pdf for example"*
We have revised language to state abortions later in pregnancy [LINES 376-381]

12. *"Wow, this quote is very powerful and implies forced pregnancy. This seems to me to go beyond not being able to access timely services and is actually referring to outright denial of care. Perhaps stronger language needed to frame this."*
We have strengthened the surrounding Results and Discussion text to more explicitly frame this account as potentially reflecting denial of care and heightened risk of compromised voluntariness, rather than describing it only as "delayed access." [LINES 376-389] and [LINES 662-671].

13. *"I don't love the framing of women's "chaotic lifestyles". It feels a little stigmatizing. While technically true, might be more appropriate to explain how women are experiencing intersecting demands and determinants of health and access upon release from prison. This removes the focus from individual behaviour choices/lifestyle…"*
We have removed "chaotic lifestyles" framing from our narrative voice throughout the manuscript and replaced it with more neutral, structurally grounded language describing post-

release instability and intersecting demands (e.g., housing insecurity, violence/unsafe relationships, substance use, legal obligations, and competing priorities) that shape access to care. Where "chaos/chaotic" appears, we have retained it only in direct quotations and have made clear it reflects participant language rather than an author framing.

14. *"Re-word to "one of the consequences on this….etc" sentence sounds incomplete as currently written"*
We have rewritten the sentence so it is grammatically complete and the causal link is explicit, clarifying what the "consequence" refers to and how it follows from the preceding point. [LINES 447-449].

DISCUSSION

15. *"What is contraceptive support? This term hasn't been used before. Simpler to say contraception?"*
Thank-you for this query. We have clarified terminology by using "contraceptive care" more consistently in the revised manuscript, including to better capture counselling, provision, and removal-related aspects of care.

16. *"Also, not sure this statement is true as currently written. Sutherland et al. (2015) did technically consider factors that may have led to low uptake in their sample. Similarly, Heggie Noonan et al. (https://www.sciencedirect.com/science/article/pii/S0277539524001572) explored how geographic factors might impede access to abortion in prison in Australia. Your study is certainly the first (as far as I can find!) to qualitatively examine in-depth multiple factors from the perspective of family planning professionals. Suggest re-wording."*
Thank-you for this observation. We have re-worded the Discussion to avoid overstating novelty; the current text now limits the claim to this being the first Australian qualitative study, to our knowledge, from the perspective of healthcare and social welfare professionals working directly with these women [LINES 639-641].

17. *"Again suggest moving away from the chaotic lifestyle framing and instead detailing some of the intense challenges faced by women upon release from prison"*
We have revised this section to avoid "chaotic lifestyle" framing and instead describe post-release instability in terms of housing insecurity, violence, substance use, and competing priorities. This has been done throughout the manuscript.

18. *"I would argue reproductive justice cannot be achieved in a prison setting. I do find the RJ framing quite weak throughout (mentioned in intro but not integrated into methodology as a theoretical framework). I would remove this statement, or expand on it. I think your study has identified numerous factors that make RJ not possible in a prison setting, such as a lack of autonomy, delay or outright denial of care, lack of culturally informed services, etc…. "*
We agree with this observation. As per our response to #8, we have not used Reproductive Justice as our primary theoretical analysis framework, but have drawn on its principles to frame the broader context of discussion on reproductive health service provision in carceral settings in both the introduction and the discussion [LINES 109-126, 718-720 and 732-733].

19. *"Several factors were identified requiring further consideration"*
We have revised the sentence for completeness and clarity. [LINES 643-644].

20. *"And perhaps speaking to incarcerated women about what outcomes and services matter to them, in this future-focused planning?"*
We have expanded the Limitations/Future Research section to clearly state that this study reports provider perspectives and that future work should include interviews with incarcerated women to understand priorities for contraception, pregnancy/parenting, and service design. We have also noted that related work is currently being explored as part of the SHAAP-2 study [LINES 792-796].

21. *"Yes, this does make RJ impossible in this setting. Suggest using this stronger language above where noted."*
We have incorporated stronger language in the Discussion explicitly noting that Reproductive Justice cannot be fully realized in this setting under the constraints described but also arguing that "this does not negate the imperative to pursue meaningful, rights-affirming improvements in contraceptive support for women who are incarcerated now" [LINES 720-725].

22. *"Can you cite a study with incarcerated men to support this statement?"*
Thank you for this comment. We have revised the wording to avoid implying that this point rests on literature about incarcerated men specifically and instead refer more cautiously to the broader literature on prison systems and women's health services. [LINES 714-718].

23. *"Re: inability to interview corrective services NSW staff, are there plans to do this in the future?"*
We have expanded Limitations/Future Research to note that corrective services staff were not included in this study and to recommend future research including corrective services perspectives alongside women's perspectives, subject to access/approval constraints. [LINES 808-813].

REVIEWER #2

ABSTRACT

1. *Minor – review for grammar in the abstract introduction as some words are used repeatedly in the same sentence*
We have copy-edited the Abstract introduction to remove repetition and improve sentence clarity (including removing duplicated phrases and tightening wording). *[LINES 31–38]*.

2. *The authors state justice involved women have "distinctive family planning needs". I struggle with this phrase and assume the authors use it to offer a neutral stance. I wonder if there is a different way to spell this out more clearly in order to set the stage for the article.*
We have revised the Abstract introduction to replace the vague phrase "distinctive family planning needs" with a more specific, evidence-linked description of what is known in the Australian literature (pregnancy and abortion/pregnancy termination histories, contraception

use patterns, and adverse birth outcomes) and how this suggests needs that are not well met by current models of care. [LINES 31-38 and 56-62]

3. *In the last sentence of the introduction, I recommend rephrasing the study objective so it is clearly stated. As it reads now, it seems the primary objective is to understand access to LARC. (see comment #7 too)*
   We have rephrased the final sentence(s) of the Abstract introduction so the study objective is clearly stated as examining how contraception is discussed, offered and accessed across the incarceration-community transition, rather than implying a LARC-specific focus. [LINES 31-38].

4. *Conclusion – I would consider expanding on the concluding sentence and go beyond attributing barriers to reproductive services all to a "lack of focus"/prioritization. The authors state several other reasons for this, including patient and clinician factors in the conclusion of the manuscript.*
   We have expanded the Abstract conclusion so it does not attribute barriers only to "lack of focus/prioritisation." The revised conclusion now explicitly reflects the multi-level constraints identified in the findings (structural constraints during incarceration, staff attitudes/biases, service integration and funding issues, and post-release circumstances), and frames the implications across system-, clinician- and patient-levels. [LINES 44-46].

INTRODUCTION

1. *Terminology*
   a. *The authors should acknowledge somewhere in the manuscript their use of gendered language and that all pregnancy capable people may not identify as "women".*
      We have added an explicit acknowledgement in the Methods that we use gendered language because participants and services were framed around "women," while noting that not all people who can become pregnant identify as women and that findings may also be relevant to pregnancy-capable people of other genders. [LINES 276-278].

   b. *I recommend being consistent throughout the article with terminology around abortion. Within the manuscript it is described as "abortion", "termination", and "pregnancy termination", stick with one.*
      We have standardised terminology throughout the manuscript by using one primary term consistently in the narrative text, while retaining participants' original wording within quotations where needed. We have also ensured the Discussion paragraph on abortion access uses consistent terminology throughout.

   c. *Instead of using the term "marginalized" consider explaining why a specific group is disadvantaged.*
      We have reduced reliance on the label "marginalised" and instead specified the intersecting structural disadvantages relevant to this population (e.g., criminalisation, poverty, trauma, violence, housing insecurity, substance use, and, critically in

Australia, over-incarceration of Aboriginal and Torres Strait Islander women within a colonial criminal-legal context). This has been done throughout the manuscript

2. *"Consider explaining why non-coercive counseling is more important in the provision of LARCs than other reversible methods of contraception. As in, because LARC require a clinician for placement and removal, patients are dependent on a clinician to start/stop these methods. (This is mentioned at other points in the manuscript but should be stated upfront".*
We have clarified this at the start of the introduction to frame the importance of these considerations when providing LARC services [LINES 66-69].

3. *"Consider using a word other than "precarious". I would argue there is no question that coercion exists within carceral institutions."*
We have incorporated this issue into a broader summary of the challenges of providing reproductive care within prison, which are inherently coercive by nature. [LINES 112-118].

4. *"Consider changing the phrase "substance abuse" to "substance use disorder"*
We have replaced stigmatising terminology with contemporary language (e.g. "substance use disorder") throughout the manuscript.

5. *"I would recommend rewording the concluding paragraph of the introduction to clearly state the objective. After reading the results section, the last sentence of the intro sounds like a secondary objective. Additionally, there is a large focus on LARC methods which to me insinuates a LARC-first approach or an overemphasis of LARC methods in this population. Is this intentional or was the goal of the study to understand perspectives about access to and provision of all forms of reversible contraception? As we continue to recognize the harms a LARC-first approach had on distinct groups whose reproductive worth has been historically devalued – including young people, racial minorities, those experiencing material poverty, and those who are incarcerated, over-emphasizing LARC feels problematic here."*
We have reworked the concluding Introduction paragraph to state one primary objective clearly, examining how contraception is discussed, offered, and accessed across custody and the post-release transition, so it does not read as a secondary or LARC-specific aim. We have also revised method discussion to avoid any LARC-first framing by presenting contraception options neutrally and foregrounding patient-centered choice, voluntariness, and discontinuation/removal access where relevant to clinician-dependent methods. [LINES 148-151].

METHODS

1. *"I recommend following the COREQ (Consolidated criteria for reporting qualitative research) checklist when reporting the methods and results. I think most of the checklist is covered but I would recommend double checking this: Tong, A., Sainsbury, P., & Craig, J. (2007). Consolidated criteria for reporting qualitative research (COREQ): a 32-item checklist for interviews and focus groups. International journal for quality in health care, 19(6), 349-357"*
We have checked reporting against COREQ and have stated that we have followed the COREQ 32-item checklist, citing Tong et al. (2007) and have provided the checklist as it applies to our study under supplementary materials [LINES 179-180].

2. *"Please elaborate on HS and their credentials and positionality as an interviewer?"*
   We have added detail on HS's credentials and positionality, including qualitative research experience relevant to incarceration and reproductive health, and clarified that HS had no clinical/managerial relationship with participants. We have also described reflexive practices used to examine assumptions during data collection/analysis. [LINES 237-240].

3. *"The authors state that "all factors were explored in the context of the provision of contraception, termination of pregnancy, and fertility treatment support" and go on to present some findings regarding abortion and no findings about fertility treatment support. My guess is that these may be explored in future analyses and manuscripts. If this is the case, I would recommend either removing the mention of abortion and fertility treatment or stating that these areas will not be discussed in this analysis and manuscript."*
   We have clarified the scope statement in Methods so it aligns with what is reported in this manuscript. Specifically, we have tightened wording to indicate that while interviews covered broader reproductive health topics, this analysis focuses on contraception (and where relevant, abortion/termination appears as contextual material linked to contraception access), and fertility treatment is not examined in this paper. [LINES 232-233].

RESULTS

1. *"Figure 1:*
   a. *Please include more description to the title to allow it to stand alone (ie include information on location (Australia, NSW) and that these "factors" are from the perspective of service workers). Similarly, spell out the abbreviations for "AMS" and "AHV" or include a legend. We have revised the Figure 1 caption so it stands alone, including the location (NSW) and clarifying that factors reflect service-provider perspectives. [LINE 313].*
   b. *Is it possible to include the factors within the red box into the larger model since they appear to lead to the same outcomes?*
      We have clarified in the analytic explanation in the Methods rather than redrawing the figure, explaining how the model domains were adapted and why these factors remained analytically distinct [LINES 266-276].
   c. *Also, I am a bit confused understanding how Figure 1 was organized after being presented with the Behavioral Model for Vulnerable Populations since it does not follow this."*
      We have added a clear explanation in the Methods describing how the Vulnerable Populations Behaviour Model guided the analytic structure and how we iteratively adapted model domains as themes developed (including relabelling "enabling factors" as "structural factors that influence access" to better reflect the data). [LINES 266-276].

2. *"Consider changing the language of "considered" and "thoughtful" to "patient-centered"*
   We have introduced "Patient-centred care" as a subheading and framing term [LINE 427], although some "thoughtful/considered" wording remains elsewhere in the manuscript [LINES 428-444 and 474-475].

3. *"The theme "interrupted fertility" is an interesting one, and I find a lot of value in the quotes. However, to suggest patients' desire for pregnancy after incarceration is a barrier to accessing contraception feels problematic. Consider contextualizing this theme differently."* We have revised the framing of this theme to avoid treating pregnancy desire as a "barrier" and instead to describe how incarceration can reshape fertility intentions and how service provision must respond in a patient-centered way (including supporting both pregnancy avoidance and pregnancy planning). We have ensured language stays descriptive and grounded in participant accounts rather than implying normative judgement. [LINES 317-319].

4. *"The terms "chaos" and "chaotic" in reference to someone's life feels harsh and critical. I do see the term is used in one of the participant quotes. I would consider changing this term to another more neutral term such as "instability" or "unpredictability" OR to place the term "chaos" in quotes to note that the term used in the theme name was drawn from the quotes"* We have revised the theme label and narrative text to use more neutral language (e.g., "instability and intersecting demands"), while retaining "chaos" only within direct quotations to reflect participant language. This has been changed for both the theme title [LINE 343] and throughout the manuscript.

5. *"While abortion is mentioned in the introduction within the context of reproductive health services, understanding how people experiencing incarceration navigate access to abortion services is not identified as an objective of the study. If the authors plan to discuss abortion in the results section – and I agree this is valuable data – I would recommend they state this in the introduction. (also see comment #10) Similarly, the authors state "for NGO staff, this high demand for termination services in the post-release period was indicative of an unmet need for contraception amongst newly released incarcerated women." This statement assumes that all individuals who seek abortion care want to use contraception which we have data to support is not the case. If the authors are going to include data on abortion services in this article, I would recommend rephrasing this statement, particularly because the quotes above and below are not explicitly reflective of this statement."*
Thank-you for highlighting this issue. We have clarified that the statements made by NGO workers about abortion services post-release are included as contextual material that illustrates participants' views about gaps in contraceptive access, while making clear that this link is participants' interpretation rather than a universal assumption. We have made this language more explicit in the Results [LINES 386-389] and Discussion [LINES 676-678].

6. *"I would hesitate to refer to LARCs as "controllable" given that a patient cannot independently control their insertion or removal"*
We have revised this wording to remove the inaccurate description of these methods as "controllable" and to distinguish more clearly between reversible methods and clinician-dependent methods [LINES 397-400].

7. *Thank-you for this suggestion. We have reviewed the themes and prioritized the theme name "Prison Health Service Priorities" over staff biases as this was felt to be the dominant idea within the theme [LINE 445].*

Thank-you for this suggestion. We have reviewed the themes and prioritized the theme name "Prison Health Service Priorities" over staff biases as this was felt to be the dominant idea within the theme *[LINE 708].*

8. *The authors include "staff biases" under "structural factors that influence access". Does it make more sense to retitle this section "enabling factors" as described in the Behavioral Model for Vulnerable Populations? In the methods section this is defined as encompassing both individual and structural characteristics"*
   We have retained the model-consistent structure but have explained why we relabelled the "enabling factors" domain as "structural factors that influence access," because participants described these conditions primarily as constraints rather than facilitators. We have more explicitly described our approach to making these adjustments to the model in the methods section *[LINES 422 – 436]*

9. *Thank-you for highlighting this. We have expanded the Discussion to interpret this quote more fully and connect it to the broader argument about continuity of culturally safe care, resourcing constraints, and funding mechanisms that limit community-controlled services' ability to engage during custody and at release. [LINES 734-745].*
   Thank-you for highlighting this. We have expanded the Discussion to interpret this quote more fully and connect it to the broader argument about continuity of culturally safe care, resourcing constraints, and funding mechanisms that limit community-controlled services' ability to engage during custody and at release. *[LINES 830 - 842].*

Discussion

10. *Thank-you for this suggestion. We have expanded the Discussion to explicitly define and apply stratified reproduction to interpret how social, economic and racial hierarchies shape whose fertility is supported versus constrained, and to link this to staff reluctance around removal, abortion barriers, and neglect of contraception in release planning, including the suggested citation [LINES 765-773].*
    Thank-you for this suggested. We have expanded the Discussion to explicitly define and apply stratified reproduction to interpret how social, economic and racial hierarchies shape whose fertility is supported versus constrained, and to link this to staff reluctance around removal, abortion barriers, and neglect of contraception in release planning, including the suggested citation *[LINES 864–874].*

11. *One approach to this is to follow my suggestion for the results section and separate "service priorities and staff biases" into two themes. Discuss the "service priorities" as is done in relation to preventive/acute care in the third ("secondly") paragraph.*
    Please see our comments above where we have taken on board these comments about restructuring the themes.

12. *Thank-you for this suggestion, we have incorporated this idea into the discussion [LINES 788-792]*
    Thank-you for this suggestion, we have incorporated this idea into the discussion *[LINES 886 – 889]*

13. *We have now included an explicit paragraph that addresses stratified reproduction in the discussion [LINES 765-773].*
    We have now included an explicit paragraph that addresses stratified reproduction in the discussion *[LINES 864 – 874]*

14. *This issue is now addressed in the stratified reproduction point that is addressed in the point above. [LINES 765-773].*
    This issue is now addressed in the stratified reproduction point that is addressed in the point above. *[LINES 864 – 874]*

15. *We have changed the term to nonconsensual sterilization, as this reflects the historical context that we are describing here [LINE 760].*
    We have changed the term to nonconsensual sterilization, as this reflects the historical context that we are describing here *[LINE 856]*

CONCLUSION

16. *We have revised the Conclusion so it does not imply staff intentions offset the impacts of bias or gatekeeping. The revised wording focuses on the structural constraints and the need to address service priorities and staff biases directly, consistent with the findings. [LINES 823-828].*
    We have revised the Conclusion so it does not imply staff intentions offset the impacts of bias or gatekeeping. The revised wording focuses on the structural constraints and the need to address service priorities and staff biases directly, consistent with the findings. *[LINES 923 - 941].*

Reviewer #3

TITLE & ABSTRACT

1. *"The title of this study somewhat misrepresents the data. The quote used in the title is essentially hearsay, it is not what the participant is saying but rather what their belief of how others working in the system may feel without any validation that is the case."*
   We have revised the title to accurately reflect the study design and perspective (service-provider views) and to avoid presenting an indirect quotation as a headline claim. We have ensured the title does not imply we measured experiences directly among incarcerated women, and we have aligned it with the provider-perspective qualitative design. *[LINES 1–7].*

INTRODUCTION

1. *"Substance abuse is an old term and would be best replaced with 'substance use' or 'substance use disorder histories'"*
   As noted above, we have now replaced "substance abuse" with contemporary, non-stigmatising terminology ("substance use" / "substance use disorder") throughout the manuscript

2. *Thank-you for picking this up. We have removed this line as you are quite right, the research doesn't exactly claim this and have maintained a broader discussion around pregnancy outcomes and contraception more generally in the introductory paragraph [LINES 56-62].*
   Thank-you for picking this up. We have removed this line as you are quite right, the research doesn't exactly claim this and have maintained a broader discussion around pregnancy outcomes and contraception more generally in the introductory paragraph *[LINES 124–132]*.

METHODS

1. *We have rewritten this sentence to clearly explain the custodial context (sentenced vs remand) in plain language so readers unfamiliar with the system can interpret the statistic correctly [LINES 186-188].*
   We have rewritten this sentence to clearly explain the custodial context (sentenced vs remand) in plain language so readers unfamiliar with the system can interpret the statistic correctly*[LINES 337 - 338]*.

2. *We have revised the section title to "Ethics Approvals" and ensured that institutional approvals are presented in the Methods [LINE 280].*
   We have revised the section title to "Ethics Approvals" and ensured that institutional approvals are presented in the Methods [LINES 224–225; 281–283]. The point about ethics content not being duplicated elsewhere is supported, but the wording about "safeguards relevant to confidentiality and voluntariness" is broader than what is explicitly stated in this section and may need softening.

3. *We have clarified the role and operating context of NGO participants (post-release support and linkage to care, and how their service role generates the perspectives reported), and we have made recruitment and role distinctions clearer between custody-based clinical roles and community-based service navigation roles. [LINES 219-224 and 286-294].*
   We have clarified the role and operating context of NGO participants (post-release support and linkage to care, and how their service role generates the perspectives reported), and we have made recruitment and role distinctions clearer between custody-based clinical roles and community-based service navigation roles. *[LINES 382 – 285]*

4. *We have strengthened the Limitations section to better explain the study's sample and scope, including the challenges of gaining access to this setting and the specialist roles of the participants interviewed. We have also clarified that these factors shape how the findings should be interpreted and underscore the need for future research that includes a broader range of perspectives. [LINES 797-813].*
   We have strengthened the Limitations section to better explain the study's sample and scope, including the challenges of gaining access to this setting and the specialist roles of the participants interviewed. We have also clarified that these factors shape how the findings should be interpreted and underscore the need for future research that includes a broader range of perspectives. [LINES 801–816].

RESULTS

1. *We have clarified the remand/sentenced composition in Methods [LINES 186-188] and have added interpretive context in the Discussion regarding how remand and short sentences can shape service continuity and decision-making [LINES 696-705].*
   We have clarified the remand/sentenced composition in Methods *[LINES 337 - 338]* and have added interpretive context where relevant in the Results *[LINES 806 – 824]* and Discussion *[LINES 968 – 974]* regarding how remand and short sentences can shape service continuity and decision-making.

2. *"The first point in the findings, "Interrupted fertility", warrants a little more clarification. The first quote is not well integrated into the description. Four factors are identified as driving this but only two of these are represented in the quotes, therefore it is unclear to the reader if these are actual findings or the authors broad interpretation of what the participants may have been saying. Only two points – 1 and 3 are discussed (as presented in the results) by the participant"*
   integrating the opening quote directly into the analytic narrative (rather than presenting it as a standalone excerpt) [LINES 320-323]
   - integrating the opening quote directly into the analytic narrative (rather than presenting it as a standalone excerpt) *[LINES 522 – 5232]*
   - tightened wording to avoid implying causal mechanisms beyond what participants reported. Practically, this meant removing/softening the earlier "four factors" framing where it was not adequately supported, and instead presenting the theme as a synthesis of what participants explicitly described, including fertility anxiety linked to menstrual disruption in custody (GP account) and post-release instability and service barriers shaping women's ability to enact reproductive intentions (NGO accounts), with clearer signposting of what is directly reported versus interpretive framing. [LINES 317-342].
   - tightened wording to avoid implying causal mechanisms beyond what participants reported. Practically, this meant removing/softening the earlier "four factors" framing where it was not adequately supported, and instead presenting the theme as a synthesis of what participants explicitly described, including fertility anxiety linked to menstrual disruption in custody (GP account) and post-release instability and service barriers shaping women's ability to enact reproductive intentions (NGO accounts), with clearer signposting of what is directly reported versus interpretive framing. *[LINES 521 - 561]*.

3. *We have clarified this wording so abortion services are discussed as contextual evidence of gaps in contraception access, without equating abortion care with contraception. [LINES 376-389].*
   We We have clarified this wording so abortion services are discussed as contextual evidence of gaps in contraception access, without equating abortion care with contraception. [LINES 376–381; 391–393].

4. *Thank-you for highlighting this and we agree. We have integrated this quote more explicitly into the Discussion by linking it to provider inaction, reproductive control, and the contrast with reluctance to remove clinician-dependent contraception [LINES 662-671].*

Thank-you for highlighting this and we agree. We have integrated this quote more explicitly into the Discussion by linking it to provider inaction, reproductive control, and the contrast with reluctance to remove clinician-dependent contraception [LINES 667–676].

5. *Thank you for this helpful comment. We have revised the sentence to improve both accuracy and clarity. In particular, we removed the inaccurate description of IUDs, implants, and injectables as "non-invasive" and "controllable," and replaced it with wording that more clearly distinguishes reversible methods from clinician-dependent methods [LINES 397-400].*
6. Thank you for this helpful comment. We have revised the sentence to improve both accuracy and clarity. In particular, we removed the inaccurate description of IUDs, implants, and injectables as "non-invasive" and "controllable," and replaced it with wording that more clearly distinguishes reversible methods from clinician-dependent methods..*[LINES 626-620]*.

We hope these revisions address the Academic Editor's requirements and reviewer concerns. Please address correspondence regarding this manuscript to the corresponding author listed on the title page.

Sincerely,

On behalf of all authors

Sincerely

Helene Smith
PhD Candidate, University of New South Wales, Australia
