# Supplementary material for: Factors that impede women’s contraceptive care needs during and immediately after incarceration in New South Wales, Australian. Qualitative perspectives of professional staff working with incarcerated women
Source: PLoS One. 2026 Aug 31;21(8):e0355277. doi: 10.1371/journal.pone.0355277 (PMC13528980; doi:10.1371/journal.pone.0355277)
Supplement: Supplementary file 5 [file pone.0355277.s005.docx]

Thank you for the opportunity to review this revised manuscript focused on access to contraception for justice-involved pregnancy capable people in Australia from the perspective of clinicians and other health professionals who provide these reproductive health services during incarceration and post-release. The revisions made by the authors are a significant enhancement to the manuscript and reflect a careful consideration of each recommendation made by the reviewers. The data presented make a meaningful contribution to the carceral reproductive health research.

My main critique of the revised manuscript is that the introduction and discussion are now both very long. Below are several minor recommendations, including some suggestions on where to remove duplicative or unnecessary information. With these minor adjustments, I think this manuscript will be suitable for publication.

Introduction

1. Lines 104-105 – consider briefly defining reproductive justice for the audience
2. Line 138 – when you say “uneven availability” do you mean “inequitable access”?
3. Line 148-149 – the term “such as Medicare or Pharmaceutical Benefits Scheme” is written twice
4. Lines 150-157 – this description of other health systems structures feels unnecessary here since the focus of the article is on Australia and the authors already give a detailed description of correctional health services there. I would consider removing this text.

Methods

5. Lines 192-198 – starting with “with the Justice Health and Forensic Mental Health Service…” – this description feels redundant to what is already stated in the introduction.
6. Line 222 – “(n=12)” does not belong in this sentence. It can be stated in the first sentence of the results section

Discussion

7. Several paragraphs appear to have been added to the discussion. While this does provide additional useful critique, the structure of the discussion is now quite drawn out. The use of first, secondly, thirdly, and fourthly, in the latter paragraphs is somewhat confusing – I had to go back and understand how these paragraphs fit together and were distinct from the proceeding ones – especially because the 2^nd^ paragraph of the discussion seems to identify and discuss the “first” factor for consideration. I would encourage the authors to review these paragraphs again and consider restructuring how they introduce them so that the discussion flows more logically and potentially removes some overlapping or redundant critiques.
