# Supplementary material for: Factors that impede women’s contraceptive care needs during and immediately after incarceration in New South Wales, Australian. Qualitative perspectives of professional staff working with incarcerated women
Source: PLoS One. 2026 Aug 31;21(8):e0355277. doi: 10.1371/journal.pone.0355277 (PMC13528980; doi:10.1371/journal.pone.0355277)
Supplement: Supplementary file 4 [file pone.0355277.s003.docx]

Thank you for the opportunity to review this manuscript focused on access to contraception for justice-involved pregnancy capable people in Australia from the perspective of clinicians and other health professionals who provide these reproductive health services during incarceration and post-release. The authors offer valuable insights through rich quotes and identify interesting themes. However, while I felt the discussion section was strong, the objectives are not clearly stated in the introduction, which leaves the rest of the manuscript difficult to follow. There is mention of abortion and fertility treatment throughout the results, but this was not a stated objective of the study. With revisions to the introduction, particularly creating clearly stated objectives, I think this paper will make a strong contribution to the literature.

I make several suggestions below that I believe will strengthen the article. Notably, the line numbers stopped after page 11 so from there on I will refer to the page numbers for my comments.

**Abstract**

1. Introduction – the authors state justice involved women have “distinctive family planning needs” – I struggle with this phrase and assume the authors use it to offer a neutral stance. I wonder if there is a different way to spell this out more clearly in order to set the stage for the article.
   a. In the last sentence of the introduction, I recommend rephrasing the study objective so it is clearly stated. As it reads now, it seems the primary objective is to understand access to LARC. (see comment #7 too)
   b. Minor – review for grammar in the abstract introduction as some words are used repeatedly in the same sentence
2. Conclusion – I would consider expanding on the concluding sentence and go beyond attributing barriers to reproductive services all to a “lack of focus”/prioritization. The authors state several other reasons for this, including patient and clinician factors in the conclusion of the manuscript.

**Introduction**

3. Terminology
   a. The authors should acknowledge somewhere in the manuscript their use of gendered language and that all pregnancy capable people may not identify as “women”.
   b. I recommend being consistent throughout the article with terminology around abortion. Within the manuscript it is described as “abortion”, “termination”, and “pregnancy termination”, stick with one.
   c. Instead of using the term “marginalized” consider explaining why a specific group is disadvantaged.
4. Line 76 – consider explaining why non-coercive counseling is more important in the provision of LARCs than other reversible methods of contraception. As in, because LARC require a clinician for placement and removal, patients are dependent on a clinician to start/stop these methods. (This is mentioned at other points in the manuscript but should be stated upfront).
5. Line 81 – consider using a word other than “precarious”. I would argue there is no question that coercion exists within carceral institutions.
6. Line 89 – consider changing the phrase “substance abuse” to “substance use disorder”
7. I would recommend rewording the concluding paragraph of the introduction to clearly state the objective. After reading the results section, the last sentence of the intro sounds like a secondary objective. Additionally, there is a large focus on LARC methods which to me insinuates a LARC-first approach or an overemphasis of LARC methods in this population. Is this intentional or was the goal of the study to understand perspectives about access to and provision of all forms of reversible contraception? As we continue to recognize the harms a LARC-first approach had on distinct groups whose reproductive worth has been historically devalued – including young people, racial minorities, those experiencing material poverty, and those who are incarcerated, over-emphasizing LARC feels problematic here.

**Methods**

8. I recommend following the COREQ (Consolidated criteria for reporting qualitative research) checklist when reporting the methods and results. I think most of the checklist is covered but I would recommend double checking this.
   a. Tong, A., Sainsbury, P., & Craig, J. (2007). Consolidated criteria for reporting qualitative research (COREQ): a 32-item checklist for interviews and focus groups. *International journal for quality in health care*, *19*(6), 349-357.
9. Please elaborate on HS and their credentials and positionality as an interviewer?
10. Lines 168-169 –The authors state that *“all factors were explored in the context of the provision of contraception, termination of pregnancy, and fertility treatment support”* and go on to present some findings regarding abortion and no findings about fertility treatment support. My guess is that these may be explored in future analyses and manuscripts. If this is the case, I would recommend either removing the mention of abortion and fertility treatment or stating that these areas will not be discussed in this analysis and manuscript.

**Results**

11. Figure 1
    a. Please include more description to the title to allow it to stand alone (ie include information on location (Australia, NSW) and that these “factors” are from the perspective of service workers). Similarly, spell out the abbreviations for “AMS” and “AHV” or include a legend.
    b. Is it possible to include the factors within the red box into the larger model since they appear to lead to the same outcomes?
    c. Also, I am a bit confused understanding how Figure 1 was organized after being presented with the Behavioral Model for Vulnerable Populations since it does not follow this.
12. Consider changing the language of “considered” and “thoughtful” to “patient-centered”
13. The theme “interrupted fertility” is an interesting one, and I find a lot of value in the quotes. However, to suggest patients’ desire for pregnancy after incarceration is a barrier to accessing contraception feels problematic. Consider contextualizing this theme differently.
14. The terms “chaos” and “chaotic” in reference to someone’s life feels harsh and critical. I do see the term is used in one of the participant quotes. I would consider changing this term to another more neutral term such as “instability” or “unpredictability” OR to place the term “chaos” in quotes to note that the term used in the theme name was drawn from the quotes.
15. Page 13 – While abortion is mentioned in the introduction within the context of reproductive health services, understanding how people experiencing incarceration navigate access to abortion services is not identified as an objective of the study. If the authors plan to discuss abortion in the results section – and I agree this is valuable data – I would recommend they state this in the introduction. (also see comment #10)
    a. Page 14 – Similarly, the authors state *“for NGO staff, this high demand for termination services in the post-release period was indicative of an unmet need for contraception amongst newly released incarcerated women.”* This statement assumes that all individuals who seek abortion care want to use contraception which we have data to support is not the case. If the authors are going to include data on abortion services in this article, I would recommend rephrasing this statement, particularly because the quotes above and below are not explicitly reflective of this statement.
16. Page 14 - I would hesitate to refer to LARCs as “controllable” given that a patient cannot independently control their insertion or removal.
17. Page 18-19 – I would consider making “staff biases” and “prison health service priorities” two separate themes as the former refers to individual bias regarding the reproductive value of people experiencing incarceration (ie stratified reproduction – exemplified by the phrase *“perceived “appropriateness” of a woman to be able to have children by staff”* on page 19) and the latter refers to institutional (carceral) priorities. (see comment #19)
    a. The authors include “staff biases” under *“structural factors that influence access”*. Does it make more sense to retitle this section *“enabling factors”* as described in the Behavioral Model for Vulnerable Populations? In the methods section this is defined as encompassing both individual and structural characteristics (lines 127-128).
18. Page 24 – this is a very interesting observation about the AMS care for people who have been involved in the carceral system. It is such a powerful quote and should be expanded on here or in the discussion.

**Discussion**

19. The first paragraph of the discussion section is very strong and summarizes the findings nicely. My suggestion for the discussion section as a whole is to expand more on the influence of societal and individual (carceral staff and clinicians) bias on contraceptive access for this population. I recommend explicitly discussing stratified reproduction *(see Harris LH, Wolfe T. Stratified reproduction, family planning care and the double edge of history. Curr Opin Obstet Gynecol. 2014 Dec 1;26(6):539-44*.) which is alluded to in both the results and discussion but not defined.
    a. Page 25-27 - One approach to this is to follow my suggestion for the results section and separate “service priorities and staff biases” into two themes. Discuss the “service priorities” as is done in relation to preventive/acute care in the third (“*secondly*”) paragraph.
       i. Perhaps consider a discussion point that if the carceral system is in the “business of rehabilitation” (for lack of a better term) there should be a focus on providing people with the resources they need to lead healthy and productive lives upon return to the community and not just prioritizing immediate needs while incarcerated
    b. Then use the fifth (“*fourthly*”) paragraph for “staff biases”. Include a discussion of stratified reproduction, perhaps suggest the need to train clinicians/staff on patient-centered contraceptive counseling, and how to address their own individual bias
       i. Page 25 - I recommend expanding on how “degendering” of women isn’t just due to lack of staff to provide services but due to societal and individual bias in regard to stratified reproduction.
20. Page 27 - Please expand on the term “structural sterilizations” or consider the term “nonconsensual sterilization”
21. In the conclusion, consider removing or rephrasing “despite the best intentions of staff” given the authors present a multitude of evidence regarding staff bias in contraceptive counseling and provision.
