# Supplementary material for: Factors that impede women’s contraceptive care needs during and immediately after incarceration in New South Wales, Australian. Qualitative perspectives of professional staff working with incarcerated women
Source: PLoS One. 2026 Aug 31;21(8):e0355277. doi: 10.1371/journal.pone.0355277 (PMC13528980; doi:10.1371/journal.pone.0355277)
Supplement: Peer review history

# Peer review: Decision Letter 0

**DOI:** 10.1371/journal.pone.0355277.r001

**Authors:** Andrea Knittel

6 Nov 2025

PONE-D-25-46905

“Why are you doing contraception? … They don't need it in jail”. Service provider views on women’s access to contraception in Australian prisons.

PLOS ONE?

Dear Dr. Smith,

Thank you for submitting your manuscript to PLOS ONE. After careful consideration, we feel that it has merit but does not fully meet PLOS ONE’s publication criteria as it currently stands. Therefore, we invite you to submit a revised version of the manuscript that addresses the points raised during the review process.

Attending to each of the concerns raised by the reviewers is necessary in order to meet the publication criteria related to clear descriptions of the research methods and also related to keeping a close linkage between the data and the conclusions.

Please submit your revised manuscript by Dec 21 2025 11:59PM. If you will need more time than this to complete your revisions, please reply to this message or contact the journal office at plosone@plos.org. When you're ready to submit your revision, log on to https://www.editorialmanager.com/pone/ and select the 'Submissions Needing Revision' folder to locate your manuscript file.

- A rebuttal letter that responds to each point raised by the academic editor and reviewer(s). You should upload this letter as a separate file labeled 'Response to Reviewers'.
- A marked-up copy of your manuscript that highlights changes made to the original version. You should upload this as a separate file labeled 'Revised Manuscript with Track Changes'.
- An unmarked version of your revised paper without tracked changes. You should upload this as a separate file labeled 'Manuscript'.

If you would like to make changes to your financial disclosure, please include your updated statement in your cover letter. Guidelines for resubmitting your figure files are available below the reviewer comments at the end of this letter.

If applicable, we recommend that you deposit your laboratory protocols in protocols.io to enhance the reproducibility of your results. Protocols.io assigns your protocol its own identifier (DOI) so that it can be cited independently in the future. For instructions see: https://journals.plos.org/plosone/s/submission-guidelines#loc-laboratory-protocols. Additionally, PLOS ONE offers an option for publishing peer-reviewed Lab Protocol articles, which describe protocols hosted on protocols.io. Read more information on sharing protocols at https://plos.org/protocols?utm_medium=editorial-email&utm_source=authorletters&utm_campaign=protocols.

We look forward to receiving your revised manuscript.

Kind regards,

Andrea K. Knittel

Academic Editor

PLOS ONE

Journal Requirements:

When submitting your revision, we need you to address these additional requirements.

1. Please ensure that your manuscript meets PLOS ONE's style requirements, including those for file naming. The PLOS ONE style templates can be found at https://journals.plos.org/plosone/s/file?id=wjVg/PLOSOne_formatting_sample_main_body.pdf and https://journals.plos.org/plosone/s/file?id=ba62/PLOSOne_formatting_sample_title_authors_affiliations.pdf

2. Your ethics statement should only appear in the Methods section of your manuscript. If your ethics statement is written in any section besides the Methods, please delete it from any other section.

3. We note that you have indicated that there are restrictions to data sharing for this study. For studies involving human research participant data or other sensitive data, we encourage authors to share de-identified or anonymized data. However, when data cannot be publicly shared for ethical reasons, we allow authors to make their data sets available upon request. For information on unacceptable data access restrictions, please see http://journals.plos.org/plosone/s/data-availability#loc-unacceptable-data-access-restrictions.

Before we proceed with your manuscript, please address the following prompts:

a) If there are ethical or legal restrictions on sharing a de-identified data set, please explain them in detail (e.g., data contain potentially identifying or sensitive patient information, data are owned by a third-party organization, etc.) and who has imposed them (e.g., a Research Ethics Committee or Institutional Review Board, etc.). Please also provide contact information for a data access committee, ethics committee, or other institutional body to which data requests may be sent.

b) If there are no restrictions, please upload the minimal anonymized data set necessary to replicate your study findings to a stable, public repository and provide us with the relevant URLs, DOIs, or accession numbers. Please see http://www.bmj.com/content/340/bmj.c181.long for guidelines on how to de-identify and prepare clinical data for publication. For a list of recommended repositories, please see https://journals.plos.org/plosone/s/recommended-repositories. You also have the option of uploading the data as Supporting Information files, but we would recommend depositing data directly to a data repository if possible.

Please update your Data Availability statement in the submission form accordingly.

4. Please provide a complete Data Availability Statement in the submission form, ensuring you include all necessary access information or a reason for why you are unable to make your data freely accessible. If your research concerns only data provided within your submission, please write "All data are in the manuscript and/or supporting information files" as your Data Availability Statement.

5. Please include captions for your Supporting Information files at the end of your manuscript, and update any in-text citations to match accordingly. Please see our Supporting Information guidelines for more information: http://journals.plos.org/plosone/s/supporting-information.

6. We note that there is identifying data in the Supporting Information file <ETHICS DOCUMENTS.zip>. Due to the inclusion of these potentially identifying data, we have removed this file from your file inventory. Prior to sharing human research participant data, authors should consult with an ethics committee to ensure data are shared in accordance with participant consent and all applicable local laws.

Data sharing should never compromise participant privacy. It is therefore not appropriate to publicly share personally identifiable data on human research participants. The following are examples of data that should not be shared:

-Name, initials, physical address

-Ages more specific than whole numbers

-Internet protocol (IP) address

-Specific dates (birth dates, death dates, examination dates, etc.)

-Contact information such as phone number or email address

-Location data

-ID numbers that seem specific (long numbers, include initials, titled “Hospital ID”) rather than random (small numbers in numerical order)

Data that are not directly identifying may also be inappropriate to share, as in combination they can become identifying. For example, data collected from a small group of participants, vulnerable populations, or private groups should not be shared if they involve indirect identifiers (such as sex, ethnicity, location, etc.) that may risk the identification of study participants.

Additional guidance on preparing raw data for publication can be found in our Data Policy (https://journals.plos.org/plosone/s/data-availability#loc-human-research-participant-data-and-other-sensitive-data) and in the following article: http://www.bmj.com/content/340/bmj.c181.long.

Please remove or anonymize all personal information (<specific identifying information in file to be removed>), ensure that the data shared are in accordance with participant consent, and re-upload a fully anonymized data set. Please note that spreadsheet columns with personal information must be removed and not hidden as all hidden columns will appear in the published file.

7. If the reviewer comments include a recommendation to cite specific previously published works, please review and evaluate these publications to determine whether they are relevant and should be cited. There is no requirement to cite these works unless the editor has indicated otherwise.

[Note: HTML markup is below. Please do not edit.]

Reviewers' comments:

Reviewer's Responses to Questions

**Comments to the Author**

1. Is the manuscript technically sound, and do the data support the conclusions?

Reviewer #1: Yes

Reviewer #2: Yes

Reviewer #3: Yes

**********

2. Has the statistical analysis been performed appropriately and rigorously?

Reviewer #1: N/A

Reviewer #2: N/A

Reviewer #3: N/A

**********

3. Have the authors made all data underlying the findings in their manuscript fully available?

The PLOS Data policy

Reviewer #1: No

Reviewer #2: No

Reviewer #3: No

**********

4. Is the manuscript presented in an intelligible fashion and written in standard English?

Reviewer #1: Yes

Reviewer #2: Yes

Reviewer #3: Yes

**********

Reviewer #1: Thank you for the opportunity to review this paper on an under-researched and important topic.

In general, I think the reproductive justice framework needs more consideration and thought to be fully realized. I have listed additional comments and suggestions below.

General note: Paper needs thorough copy editing for grammar, particularly use of commas when not appropriate.

Abstract

Line 32-33 - I suggest re-wording your description of the sample since actually only 8 participants were clinicians. I certainly see the value of interviewing other people who play a role in the provision of healthcare, but would not consider them to be “providing healthcare services”. Perhaps professionals working in family planning? Or similar wording.

Introduction

Line 65 - Can you state some of the birth outcomes?

Line 68 - And including access to abortion?

Line 80-81 - I would suggest stronger language here. Beyond questions of coercion becoming precarious, I would argue the prison environment is inherently coercive. If you are using an RJ framing here, would also suggest a few lines on how the prison environment interrupts and systematically separates families, in direct conflict with RJ tenet to be able to parent in safe and sustainable communities free from violence. I also think this introduction needs some reference to the over-incarceration of Aboriginal and Torres Strait Islander women in Australia, and the colonial context of the prison system, which has huge implications for reproductive justice.

Line 102-105 - In light of the lack of studies in the Australian context, can you look to analogous contexts internationally where there has been work done on family planning services in prison? E.g. Martha Paynter (Canada), Carolyn Sufrin (USA). You could say studies conducted in other carceral settings have found xxx… however an Australian study is needed because of (unique context of Medicare, PBS etc.).

Methods

Line 147 - Suggest briefly defining an Aboriginal health worker for non-Australian audience who may not have an equivalent role in their context

Line 149-150 - Do they submit the request directly to nursing staff? Or is it first submitted to a guard, who then submits to nursing staff? This has implications for patient confidentiality and ethics (if first submitted to officer/guard, which has been noted in Canadian & American studies)

Line 171 - Since you state using a theoretical framework of reproductive justice, how was this considered or included in your analysis process? The RJ framing gets a little lost past the introduction.

Results

General note: I suggest referring to the terminology guide published by the Australian National Network of Incarcerated Women and Girls which does not recommend the term ‘justice-involved’: https://thenationalnetwork.com.au/abolition-resources/

Line 195-197 - Not sure this is needed.

Note that line numbering stops after the figure. Please add line numbering in future submission to facilitate review.

Page 14 - Please refer to clinical language guidelines re:abortion and avoid ‘later term abortion’: https://www.acog.org/contact/media-center/abortion-language-guide or https://www.childrenbychoice.org.au/wp-content/uploads/2023/01/Language-Guide-2022.pdf for example

Page 14- Wow, this quote is very powerful and implies forced pregnancy. This seems to me to go beyond not being able to access timely services and is actually referring to outright denial of care. Perhaps stronger language needed to frame this.

Page 15- I don’t love the framing of women’s “chaotic lifestyles”. It feels a little stigmatizing. While technically true, might be more appropriate to explain how women are experiencing intersecting demands and determinants of health and access upon release from prison. This removes the focus from individual behaviour choices/lifestyle…

Page 18 - Re-word to “one of the consequences on this….etc” sentence sounds incomplete as currently written.

Discussion

Line 2 - What is contraceptive support? This term hasn’t been used before. Simpler to say contraception?

See note above re: justice-involved

Also, not sure this statement is true as currently written. Sutherland et al. (2015) did technically consider factors that may have led to low uptake in their sample. Similarly, Heggie Noonan et al. (https://www.sciencedirect.com/science/article/pii/S0277539524001572) explored how geographic factors might impede access to abortion in prison in Australia. Your study is certainly the first (as far as I can find!) to qualitatively examine in-depth multiple factors from the perspective of family planning professionals. Suggest re-wording.

Page 25- Again suggest moving away from the chaotic lifestyle framing and instead detailing some of the intense challenges faced by women upon release from prison.

Page 25- I would argue reproductive justice cannot be achieved in a prison setting. I do find the RJ framing quite weak throughout (mentioned in intro but not integrated into methodology as a theoretical framework). I would remove this statement, or expand on it. I think your study has identified numerous factors that make RJ not possible in a prison setting, such as a lack of autonomy, delay or outright denial of care, lack of culturally informed services, etc….

Page 25 - “Several factors were identified requiring further consideration” Incomplete sentence

Page 25 second paragraph - and perhaps speaking to incarcerated women about what outcomes and services matter to them, in this future-focused planning?

Page 26 first paragraph- Yes, this does make RJ impossible in this setting. Suggest using this stronger language above where noted.

Page 26 first paragraph - can you cite a study with incarcerated men to support this statement?

Page 28 - Re: inability to interview corrective services NSW staff, are there plans to do this in the future?

Reviewer #2: All comments are included in the attached document. Overall this is an interesting and important analysis however I do think revisions are needed to improve the readability and message of the manuscript.

Reviewer #3: Thank you for the opportunity to review this study. Overall, it is well written and raises some important points regarding women's health, reproduction and contraception in the (NSW) prison system. However, there are several points which need further consideration. These are I have outlined below.

•The title of this study somewhat misrepresents the data. The quote used in the title is essentially hearsay, it is not what the participant is saying but rather what their belief of how others working in the system may feel without any validation that is the case.

•Introduction - Page 4 line 89, substance abuse is an old term and would be best replaced with ‘substance use’ or ‘substance use disorder histories’.

•The authors claim that long-acting reversible contraception has a higher uptake among women in prison then other women, I am not sure this is correct and only one grey literature source is used to support it (ref 3) which to my reading does not actually support claim. The statement warrants clearer validation or classification and if retained may need a definition of LARC, noting that depot injections are not necessarily consider LARC.

•Methods - page 6 line 133, second sentence, “Nearly 50% were sentenced” - what does this mean? Presumably it means that read the remainder were on remand, this needs to be explained so that it can be readily interpreted by a broader audience.

•Under the title “Ethical considerations”, there is simply a statement of institutional approvals and no discussion of actual ethical considerations. Either the title of the section should be changed or some discussion of the ethical issues should be outlined.

•The methods need a little bit more clarity with regard to the role of NGO workers - I assume that these operating the immediate post-release periods and not directly within the custodial setting? This needs to be made clearer.

•The sample size is very small. This has been explained in the limitations, and given those limitations is probably adequate for the study. I would note, however, that the length of time it takes to receive ethics or institutional approval is a research process and practical limitation, I do not think it is reasonable to claim that because it would have taken longer to conduct the research it was not done. I appreciate the challenges here, but if those challenges limit the ability to conduct the research, then the study’s scientific validity needs to be considered. I would argue it is only reasonable justification if it is not feasible to undertake or attempts were unsuccessful. In other words, I would suggest the expression of these points warrant consideration.

•Given the very high number of women who were on remand, if I have interpreted correctly, some further discussion is required around what this means for the meaningfully provision of any type of healthcare service. This is touched on in the discussion but not really given adequate depth. Further, there appears to be no discussion of issues such as short sentencing, unanticipated transfers and other matters which may seriously impede the women’s ability to both ask for and access health care services in the custodial setting or for health care providers to adequately provide those services. I was surprised that this was absent from the discussion as it is a major structural barrier, was this considered or highlighted in any detail by the participants?

•The first point in the findings, “Interrupted fertility”, warrants a little more clarification. The first quote is not well integrated into the description. Four factors are identified as driving this but only two of these are represented in the quotes, therefore it is unclear to the reader if these are actual findings or the authors broad interpretation of what the participants may have been saying. Only two points – 1 and 3 are discussed (as presented in the results) by the participants.

•Page 13, author states that “NGO staff stated that they often witnessed the consequences of not being able to access contraception in the community, namely demand for support in accessing pregnancy termination services." Termination services are distinct contraception services. This confirmation needs to be outlined more clearly.

•There is a very interesting quote on page 14 about women attempting to access termination services. This is important but is not well integrated into the findings and completely absent in the discussion. This is important because it points to contradictions in the system and potentially among providers when contrasted with other points made about reluctance to remove long-acting contraception and attempts to control women’s fertility. Arguably a termination is much more urgent than contraception removal but a reluctance to act or meaningfully engage with a woman’s request is deeply problematic on both counts. These points are insightful with regard to reproductive control when considered together and warrant more detailed discussion.

•Last sentence on page 14 “participants observed a high demand for non-invasive inflexible methods including non-permanent, controllable contraception such as intra uterine devices…” this is confusing as IUDs are invasive, as are several other methods listed including contraceptive implants and injectables.

**********

what does this mean?). If published, this will include your full peer review and any attached files.

If you choose “no”, your identity will remain anonymous but your review may still be made public.

**Do you want your identity to be public for this peer review?**  For information about this choice, including consent withdrawal, please see our Privacy Policy

Reviewer #1: No

Reviewer #2: No

Reviewer #3: No

**********

[NOTE: If reviewer comments were submitted as an attachment file, they will be attached to this email and accessible via the submission site. Please log into your account, locate the manuscript record, and check for the action link "View Attachments". If this link does not appear, there are no attachment files.]

To ensure your figures meet our technical requirements, please review our figure guidelines: https://journals.plos.org/plosone/s/figures

You may also use PLOS’s free figure tool, NAAS, to help you prepare publication quality figures: https://journals.plos.org/plosone/s/figures#loc-tools-for-figure-preparation.

NAAS will assess whether your figures meet our technical requirements by comparing each figure against our figure specifications.

Attachment Submitted filename: PLOS One review.pdf

Attachment Submitted filename: PONE-D-25-46905.docx

# Author comment: Author response to Decision Letter 1

**DOI:** 10.1371/journal.pone.0355277.r002

14 May 2026

We have reviewed all feedback and made the recommended changes as requested. These are listed below, alongside a detailed outline of how each point has been addressed.

Please note that the line references below refer to the line reference in the TRACKED CHANGES document.

Academic editor / journal requirements:

1. “Please ensure that your manuscript meets PLOS ONE's formatting requirements. PLOS ONE has a manuscript format similar to the sample papers listed below”

Thank-you for providing these examples. We have checked formatting against the PLOS ONE title/affiliations and main-body samples and updated the title page and manuscript layout accordingly

2. “Your ethics statement should only appear in the Methods section and not elsewhere. Please ensure your ethics statement only appears in the methods section as is required.”

We have reviewed the full manuscript and ensured that ethics information is stated in the Methods section only under Ethics Approvals [LINE 280]

3. “We note that you have indicated that there are restrictions to data access. Please include the following information in the Data Availability statement in the submission form accordingly”

We have updated the Data Availability Statement in the PLOS submission form to address restrictions, what data are restricted, and where requests may be sent

4. “Please include captions for your Supporting Information files. This information should be included in the submission form on the Attach Files page. In addition, please ensure that your file names use the following format and are cited properly in the manuscript: S1 File, S1 Table, S1 Fig, etc.”

We have ensured Supporting Information files use the required naming conventions for the figure provided. and that it has an appropriate caption.

5. “We note that there is identifying data in the Supporting Information file provided. Please remove identifying information and resubmit. Note that all columns in Supporting Information will appear in the published file. Therefore, please ensure that no columns are hidden as all hidden columns will appear in the published file.”

We reviewed the Supporting Information files for identifying content and hidden columns. The files visible to us do not appear to contain identifying participant information beyond investigator names on ethics documents. If the editorial comment refers to another supporting file, we would be grateful for clarification so we can address it directly. Should this comment be referring to another file, please do let us know so we can address this.

6. “If the reviewer comments include a recommendation to cite additional works, please review and consider such recommendations, and incorporate them into the manuscript if appropriate.”

We have reviewed all citation recommendations and updated references where relevant and appropriate throughout the manuscript

Reviewer #1

ABSTRACT:

1. I suggest re-wording your description of the sample to note not all participants were clinicians. It currently suggests all participants are “provide healthcare services to women”. You can broaden this a little. Only 8 participants were clinicians, I suggest similar wording.”

Thank-you for pointing this out. We have revised both the Abstract and Methods to accurately reflect the composition of the sample and avoid implying that all participants were clinicians. Specifically, we revised the wording to make clear that the sample included both clinical and non-clinical participants working during incarceration and/or the immediate post-release period, including healthcare providers and participants in case-management roles. This change is reflected in the Abstract Methods [LINES 36-38], and the distribution of roles (including that 8 participants were clinicians) is clarified in the Participant characteristics section [LINES 286-297].

2. Can you state some of the birth outcomes?

We have specified the adverse birth outcomes rather than referring to “poorer birth outcomes” generally; the Introduction now explicitly notes increased likelihood of preterm birth, low birth weight, and neonatal/intensive care stay, alongside infant mortality differences. [LINES 56-62]

3. “And including access to abortion?”

We have clarified in the introduction that women with a history of incarceration report accessing abortion services more than women in the general community. [LINES 56-57] As there is scant evidence on how easy it is for these same women to access abortion in the community, we have kept the point in the introduction to where the existing research lies: reporting higher number. We then address the lack of evidence and comparable evidence around women from lower socio-economic backgrounds in the discussion, in light of the findings where some participants believed that women struggled to access timely abortion care in the community [LINES 662-678].

4. “I would suggest stronger language here. Beyond questions of coercion becoming precarious, I would argue the prison environment is inherently coercive. If you are using an RJ framing here, would also suggest a few lines on how the prison environment interrupts and systematically separates families, in direct conflict with RJ tenet to be able to parent in safe and sustainable communities free from violence. I also think this introduction needs some reference to the over-incarceration of Aboriginal and Torres Strait Islander women in Australia, and the colonial context of the prison system, which has huge implications for reproductive justice”

Thank-you for highlighting this. We have revised the introduction text to state explicitly that carceral settings are inherently coercive and constrain autonomy, and we have added text on systematic family separation/conflict with the RJ tenet of parenting in safe/sustainable communities. We have also added a paragraph on over-incarceration of Aboriginal and Torres Strait Islander women and the colonial context and implications for RJ. [LINES 112-126]

5. “In light of the lack of studies in the Australian context, can you look to analogous contexts internationally where there has been work done on family planning services in prison? E.g. Martha Paynter (Canada), Carolyn Sufrin (USA). You could say studies conducted in other carceral settings have found xxx… however an Australian study is needed because of (unique context of Medicare, PBS etc.)”

We have rewritten this section so the logic is explicit rather than implied. Specifically, we have:

• clarified the evidence gap (limited Australian custody-focused contraception research) [LINES 128-129]

• explained why international models (primarily North American) may not transfer directly, and stated the key structural reason for this difference in Australia, primarily due to different funding mechanisms that fundamentally impact how services are delivered [LINES 130-146]

METHODS:

6. “Suggest briefly defining an Aboriginal health worker for non-Australian audience who may not have an equivalent role in their context”

We have added a brief definition of an Aboriginal Health Worker/Practitioner in the Methods where this role is first mentioned, describing it as an Indigenous-specific health workforce role that provides culturally safe care and supports engagement, navigation and continuity between clients and health services (often including health promotion, advocacy and liaison). [LINES 203-207].

7. “Do they submit the request directly to nursing staff? Or is it first submitted to a guard, who then submits to nursing staff? This has implications for patient confidentiality and ethics (if first submitted to officer/guard, which has been noted in Canadian & American studies)”

We have clarified the actual pathway for requesting health services in custody so readers can see when confidentiality may be protected versus when it may be compromised. In the Study Setting/Access description, we now specify that women must submit a written request and that this request may be (a) handed directly to nursing staff, (b) handed to correctional staff, or (c) placed in a request box accessed and triaged directly by nursing staff. We have included this detail because the involvement of correctional staff in request submission has clear implications for privacy and aligns with confidentiality concerns raised in international prison-health literature. [LINES 208-212].

8. “Since you state using a theoretical framework of reproductive justice, how was this considered or included in your analysis process? The RJ framing gets a little lost past the introduction”

Thank-you for this observation. In the Introduction, we have clarified that Reproductive Justice (RJ) is not used as our primary theoretical analysis framework, instead we draw on RJ principles to frame the broader context of discussion of reproductive health service provision in carceral settings, and to highlight the inherent contradictions this creates for women’s reproductive autonomy [LINES 109-126]. For the analytical framework, we now state more explicitly in the Methods that our analysis was initially guided by the Vulnerable Populations Behaviour Model and then iteratively adapted as themes developed, including a specific adaptation to better capture organisational/system constraints (reframing “enabling factors” as “structural factors influencing access”). [LINES 179-180 and 266-276]. In the Discussion, we have strengthened integration of RJ by explicitly linking our findings on constrained autonomy, gatekeeping, and discontinuity across custody-community transitions to RJ principles, and we have adopted stronger language about the impossibility of fully realising RJ under the constraints described. [LINES 718-720 and 732-733].

RESULTS:

9. “General note: I suggest referring to the terminology guide published by the Australian National Network of Incarcerated Women and Girls which does not recommend the term ‘justice-involved’: https://thenationalnetwork.com.au/abolition-resources/”

Thank-you for providing this resource. We have adapted our language throughout to describe either women with a history of incarceration, or who are incarcerated. This has been done throughout the manuscript.

10. “Note that line numbering stops after the figure. Please add line numbering in future submission to facilitate review.”

We apologise for this, this has now been rectified throughout the manuscript

11. “Please refer to clinical language guidelines re:abortion and avoid ‘later term abortion’: https://www.acog.org/contact/media-center/abortion-language-guide or https://www.childrenbychoice.org.au/wp-content/uploads/2023/01/Language-Guide-2022.pdf for example”

We have revised language to state abortions later in pregnancy [LINES 376-381]

12. “Wow, this quote is very powerful and implies forced pregnancy. This seems to me to go beyond not being able to access timely services and is actually referring to outright denial of care. Perhaps stronger language needed to frame this.”

We have strengthened the surrounding Results and Discussion text to more explicitly frame this account as potentially reflecting denial of care and heightened risk of compromised voluntariness, rather than describing it only as “delayed access.” [LINES 376-389] and [LINES 662-671].

13. “I don’t love the framing of women’s “chaotic lifestyles”. It feels a little stigmatizing. While technically true, might be more appropriate to explain how women are experiencing intersecting demands and determinants of health and access upon release from prison. This removes the focus from individual behaviour choices/lifestyle…”

We have removed “chaotic lifestyles” framing from our narrative voice throughout the manuscript and replaced it with more neutral, structurally grounded language describing post-release instability and intersecting demands (e.g., housing insecurity, violence/unsafe relationships, substance use, legal obligations, and competing priorities) that shape access to care. Where “chaos/chaotic” appears, we have retained it only in direct quotations and have made clear it reflects participant language rather than an author framing.

14. “Re-word to “one of the consequences on this….etc” sentence sounds incomplete as currently written”

We have rewritten the sentence so it is grammatically complete and the causal link is explicit, clarifying what the “consequence” refers to and how it follows from the preceding point. [LINES 447-449].

DISCUSSION

15. “What is contraceptive support? This term hasn’t been used before. Simpler to say contraception?”

Thank-you for this query. We have clarified terminology by using “contraceptive care” more consistently in the revised manuscript, including to better capture counselling, provision, and removal-related aspects of care.

16. “Also, not sure this statement is true as currently written. Sutherland et al. (2015) did technically consider factors that may have led to low uptake in their sample. Similarly, Heggie Noonan et al. (https://www.sciencedirect.com/science/article/pii/S0277539524001572) explored how geographic factors might impede access to abortion in prison in Australia. Your study is certainly the first (as far as I can find!) to qualitatively examine in-depth multiple factors from the perspective of family planning professionals. Suggest re-wording.”

Thank-you for this observation. We have re-worded the Discussion to avoid overstating novelty; the current text now limits the claim to this being the first Australian qualitative study, to our knowledge, from the perspective of healthcare and social welfare professionals working directly with these women [LINES 639-641].

17. “Again suggest moving away from the chaotic lifestyle framing and instead detailing some of the intense challenges faced by women upon release from prison”

We have revised this section to avoid “chaotic lifestyle” framing and instead describe post-release instability in terms of housing insecurity, violence, substance use, and competing priorities. This has been done throughout the manuscript.

18. “I would argue reproductive justice cannot be achieved in a prison setting. I do find the RJ framing quite weak throughout (mentioned in intro but not integrated into methodology as a theoretical framework). I would remove this statement, or expand on it. I think your study has identified numerous factors that make RJ not possible in a prison setting, such as a lack of autonomy, delay or outright denial of care, lack of culturally informed services, etc…. “

We agree with this observation. As per our response to #8, we have not used Reproductive Justice as our primary theoretical analysis framework, but have drawn on its principles to frame the broader context of discussion on reproductive health service provision in carceral settings in both the introduction and the discussion [LINES 109-126, 718-720 and 732-733].

19. “Several factors were identified requiring further consideration”

We have revised the sentence for completeness and clarity. [LINES 643-644].

20. “And perhaps speaking to incarcerated women about what outcomes and services matter to them, in this future-focused planning?”

We have expanded the Limitations/Future Research section to clearly state that this study reports provider perspectives and that future work should include interviews with incarcerated women to understand priorities for contraception, pregnancy/parenting, and service design. We have also noted that related work is currently being explored as part of the SHAAP-2 study [LINES 792-796].

21. “Yes, this does make RJ impossible in this setting. Suggest using this stronger language above where noted.”

We have incorporated stronger language in the Discussion explicitly noting that Reproductive Justice cannot be fully realized in this setting under the constraints described but also arguing that “this does not negate the imperative to pursue meaningful, rights-affirming improvements in contraceptive support for women who are incarcerated now” [LINES 720-725].

22. “Can you cite a study with incarcerated men to support this statement?”

Thank you for this comment. We have revised the wording to avoid implying that this point rests on literature about incarcerated men specifically and instead refer more cautiously to th

Attachment Submitted filename: Response to Reviewers.pdf

# Peer review: Decision Letter 1

**DOI:** 10.1371/journal.pone.0355277.r003

**Authors:** Andrea Knittel

21 Jul 2026

Factors that impede women’s contraceptive care needs during and immediately after incarceration in New South Wales, Australian. Qualitative perspectives of professional staff working with incarcerated women.

PONE-D-25-46905R1

Dear Dr. Smith,

We’re pleased to inform you that your manuscript has been judged scientifically suitable for publication and will be formally accepted for publication once it meets all outstanding technical requirements.

Within one week, you’ll receive an e-mail detailing the required amendments. When these have been addressed, you’ll receive a formal acceptance letter and your manuscript will be scheduled for publication.

An invoice will be generated when your article is formally accepted. Please note, if your institution has a publishing partnership with PLOS and your article meets the relevant criteria, all or part of your publication costs will be covered. Please make sure your user information is up-to-date by logging into Editorial Manager at Editorial Manager® and clicking the ‘Update My Information' link at the top of the page. For questions related to billing, please contact billing support.

If your institution or institutions have a press office, please notify them about your upcoming paper to help maximize its impact. If they’ll be preparing press materials, please inform our press team as soon as possible -- no later than 48 hours after receiving the formal acceptance. Your manuscript will remain under strict press embargo until 2 pm Eastern Time on the date of publication. For more information, please contact onepress@plos.org.

Kind regards,

Andrea K. Knittel

Academic Editor

PLOS One

Additional Editor Comments (optional):

Reviewers' comments:

Reviewer's Responses to Questions

**Comments to the Author**

Reviewer #1: All comments have been addressed

Reviewer #2: All comments have been addressed

**********

2. Is the manuscript technically sound, and do the data support the conclusions?

Reviewer #1: Yes

Reviewer #2: Yes

**********

3. Has the statistical analysis been performed appropriately and rigorously?

Reviewer #1: N/A

Reviewer #2: N/A

**********

4. Have the authors made all data underlying the findings in their manuscript fully available?

The PLOS Data policy

Reviewer #1: Yes

Reviewer #2: Yes

**********

5. Is the manuscript presented in an intelligible fashion and written in standard English?

Reviewer #1: Yes

Reviewer #2: Yes

**********

Reviewer #1: Thank you for your work and attention to address the reviewer comments. I have no further comments for the authors.

Reviewer #2: I have attached my minor comments regarding the revisions. Overall, the revisions made by the authors are a significant enhancement to the manuscript and reflect a careful consideration of each recommendation made by the reviewers. The results is that some sections are now very lengthy, and I included some comments on areas where this can be addressed.

**********

what does this mean?). If published, this will include your full peer review and any attached files.

If you choose “no”, your identity will remain anonymous but your review may still be made public.

**Do you want your identity to be public for this peer review?**  For information about this choice, including consent withdrawal, please see our Privacy Policy

Reviewer #1: No

Reviewer #2: No

**********

Attachment Submitted filename: PONE-D-25-46905_Revision.docx

# Editor report: Acceptance letter

**DOI:** 10.1371/journal.pone.0355277.r004

**Authors:** Andrea Knittel

PONE-D-25-46905R1

PLOS One

Dear Dr. Smith,

I'm pleased to inform you that your manuscript has been deemed suitable for publication in PLOS One. Congratulations! Your manuscript is now being handed over to our production team.

At this stage, our production department will prepare your paper for publication. This includes ensuring the following:

* All references, tables, and figures are properly cited

* All relevant supporting information is included in the manuscript submission,

* There are no issues that prevent the paper from being properly typeset

You will receive further instructions from the production team, including instructions on how to review your proof when it is ready. Please keep in mind that we are working through a large volume of accepted articles, so please give us a few days to review your paper and let you know the next and final steps.

Lastly, if your institution or institutions have a press office, please let them know about your upcoming paper now to help maximize its impact. If they'll be preparing press materials, please inform our press team within the next 48 hours. Your manuscript will remain under strict press embargo until 2 pm Eastern Time on the date of publication. For more information, please contact onepress@plos.org.

You will receive an invoice from PLOS for your publication fee after your manuscript has reached the completed accept phase. If you receive an email requesting payment before acceptance or for any other service, this may be a phishing scheme. Learn how to identify phishing emails and protect your accounts at https://explore.plos.org/phishing.

If we can help with anything else, please email us at customercare@plos.org.

Thank you for submitting your work to PLOS One and supporting open access.

Kind regards,

PLOS ONE Editorial Office Staff

on behalf of

Dr. Andrea K. Knittel

Academic Editor

PLOS One
